# Fragment based group QSAR and molecular dynamics mechanistic studies on arylthioindole derivatives targeting the α-β interfacial site of human tubulin

**DOI:** 10.1186/1471-2164-15-S9-S3

**Published:** 2014-12-08

**Authors:** Chetna Tyagi, Ankita Gupta, Sukriti Goyal, Jaspreet Kaur Dhanjal, Abhinav Grover

**Affiliations:** 1 School of Biotechnology, Jawaharlal Nehru University, New Delhi, India - 110067; 2 Department of Biotechnology, Delhi Technological University, New Delhi, India -110042; 3 Apaji Institute of Mathematics & Applied Computer Technology, Banasthali University, Tonk, Rajasthan, India - 304022

**Keywords:** Cancer, arylthioindole, QSAR, tubulin, G-QSAR, inhibitor

## Abstract

**Background:**

A number of microtubule disassembly blocking agents and inhibitors of tubulin polymerization have been elements of great interest in anti-cancer therapy, some of them even entering into the clinical trials. One such class of tubulin assembly inhibitors is of arylthioindole derivatives which results in effective microtubule disorganization responsible for cell apoptosis by interacting with the colchicine binding site of the β-unit of tubulin close to the interface with the α unit. We modelled the human tubulin β unit (chain D) protein and performed docking studies to elucidate the detailed binding mode of actions associated with their inhibition. The activity enhancing structural aspects were evaluated using a fragment-based Group QSAR (G-QSAR) model and was validated statistically to determine its robustness. A combinatorial library was generated keeping the arylthioindole moiety as the template and their activities were predicted.

**Results:**

The G-QSAR model obtained was statistically significant with r2 value of 0.85, cross validated correlation coefficient q2 value of 0.71 and pred_r2 (r2 value for test set) value of 0.89. A high F test value of 65.76 suggests robustness of the model. Screening of the combinatorial library on the basis of predicted activity values yielded two compounds HPI (predicted pIC50 = 6.042) and MSI (predicted pIC50 = 6.001) whose interactions with the D chain of modelled human tubulin protein were evaluated in detail. A toxicity evaluation resulted in MSI being less toxic in comparison to HPI.

**Conclusions:**

The study provides an insight into the crucial structural requirements and the necessary chemical substitutions required for the arylthioindole moiety to exhibit enhanced inhibitory activity against human tubulin. The two reported compounds HPI and MSI showed promising anti cancer activities and thus can be considered as potent leads against cancer. The toxicity evaluation of these compounds suggests that MSI is a promising therapeutic candidate. This study provided another stepping stone in the direction of evaluating tubulin inhibition and microtubule disassembly degeneration as viable targets for development of novel therapeutics against cancer.

## Background

Tubulin inhibition has been considered a viable avenue for drug development in cancer management for a very long time [1-4]. It plays a major role in the formation of microtubule assembly. Microtubules are polar cytoskeletal filaments that either takes part in the formation of mitotic spindle and interphase networks or more complex formations like ciliary axoneme, centrioles and basal bodies [5-7]. Their disorientation, either through inhibition of tubulin polymerization or by blocking microtubule assembly, leads to metaphase arrest of cell division [8-10]. Colchicine, the most commonly used metaphase arrest agent, has a similar mode of action [11]. Apart from these, combretastatin A-4 and the *Catharanthus *alkaloids vincristine and vinblastine are also tubulin assembly inhibitors [12,13]. Combretastatin A-4 phosphate was found to block blood flow in capillaries supporting cancerous cells by completely disrupting the endothelial cell integrity leading to rapid tumor cell death [14,15]. These inhibitors lead to microtubule disorganization and renders cell apoptosis [16]. However, the use of these previously identified tubulin inhibitors were restricted on account of excessive toxicity, drug resistance and scarce bioavailability [17-20]. Thus, development of novel tubulin assembly inhibitors is the need of the hour.

A variety of chemical compounds are known to bind to the α-β interfacial site of tubulin. These chemical compounds can be grouped under following classes (a) group targeting the lumenal site of α subunit i.e. taxol (b) group stabilizing microtubule polymerization i.e. vinblastine and (c) group targeting α-β interfacial site of tubulin that leads to cell apoptosis i.e. colchicine [21-23]. We are interested in the colchicine-like group of compounds for cancer management. The present study is based on a class of tubulin-inhibitors known as arylthioindoles that bind to the colchicine-binding site of β-tubulin close to the interface with α-tubulin. All the previous studies undertaken in this respect involved *Bos taurus* tubulin protein assembly comprising of chains A, B, C, D and E of which A and C belong to the α unit and B and D belong to the β unit [24,25]. Many previously known tubulin inhibitors consisted of the indole nucleus in the core structure and hence are touted to be one of the most potent compounds against tubulin polymerization [17,26]. Arylthioindoles were also found to be potent inhibitors of the growth of MCF-7 human breast carcinoma cells [19].

Development of accurate and time effective drug discovery techniques is the need of the hour to propagate search for novel anti-tumorals. Exploiting one of the recent and innovative approaches known as fragment based group quantitative structure activity relationship (G-QSAR) [27], the relationship between different molecular fragments and their biological activity can be correlated and studied in detail giving site-specific clues for modification [28]. Such modifications in terms of substituents added or removed lead to activity enhancement. The knowledge of such modifications is based on various molecular descriptors calculated and used for G QSAR model construction. Various such studies have been reported and have proved to be very useful [29-31], many of them to discover cancer therapeutics [32]. These descriptors are calculated for various fragments defined by the user. The optimal subset of descriptors is chosen by any one of the variable selection methods which are most likely to describe all the physicochemical properties of the congeneric series required for their biological activity. Thus, it gives a better idea about which substitution site should be populated with which particular substituent for activity enhancement [33].

In this study, we search for tubulin inhibitors having a similar binding mode as that of colchicine at the α-β interfacial site. Arylthioindole moiety is known to be a potent anti-tubulin agent and has been studied very often for its anti-cancer properties but drug toxicity and less bioavailability were the problems encountered [34]. In order to exploit this avenue further, we created a robust, accurate and predictive G-QSAR model to enhance our understanding of arylthioindole derivatives as anti-cancer compounds in terms of structural requirements needed for drug development. Based on the G-QSAR model, we identified novel therapeutic compounds with improved tubulin assembly inhibition and potent anticancer activities. The compounds were validated for their interactive properties with the colchicine binding site of tubulin by docking analysis. The resultant top two compounds were also evaluated for their absorption, distribution, metabolism, excretion and toxicity (ADMET) properties.

## Materials and methods

### Compound dataset for model development

In this study, a congeneric series of 42 tubulin inhibitors belonging to the arylthioindole class of compounds [20,35] were selected for G-QSAR model development. Due to higher root-mean-square-deviation (RMSD) values, 6 compounds (6b, 15, 20b, 24, 28 and 41b in Additional file 1) were rejected and the model was built using 36 arylthioindole derivatives. The 2D structures were drawn using Marwin Sketch [36]. They were converted to 3D by *Vlife Engine platform *of VLifeMDS and later energy minimized using the force field batch minimization utility with default parameters [37]. These optimized compounds were finally used for G-QSAR model development. The template is used as a structural moiety common to all the compounds of the congeneric dataset with the substitution sites marked by unknown (dummy) atoms. In this case there were six substitution sites, depicted by R1 to R6 in the template.

### Computation of Molecular Descriptors

After selecting the congeneric set of molecules and the template for model development, physicochemical descriptors were calculated using the *calculate descriptors *dialog of the G-QSAR module in VLife MDS. The software provides a large number of molecular descriptors belonging to the 2-dimensional and 3-dimensional classes. In case of Group QSAR, 2D descriptors are selected. All descriptors were chosen except dipole moment, electrostatic, semi-empirical and hydrophobicity as they are 3D descriptors and Information Theory-based descriptors. In G-QSAR, a variety of descriptors along with alignment independent descriptors for the fragments were calculated. Of the total 1027 descriptors calculated for all the six substitution sites, 298 were selected and the invariable descriptors were removed. Invariable descriptors are those which have same quantitative value for each data-point and thus should be discarded for their inefficacy in G-QSAR model development.

### G-QSAR model development

For data selection using the *advanced data selection wizard*, the training and test set compounds were chosen after selecting the activity pIC50 as dependent variable and all the calculated descriptors as independent variables. The test set including the compounds 10, 14, 18, 21, 22, 27b, 29, 30, 31, 35b (available in Additional file 1) was selected using *random selection method *with 80% compounds in the training set. After calculating the unicolumn statistics for the selected training and test set molecules, the *stepwise-forward variable selection *method along with PLS (Partial Least Square) [38] as the regression method for building the model was chosen through the *variable selection and model building wizard*. Keeping the cross-correlation limit set at 0.5, Ftest In at 4.0, Ftest Out at 3.0, term selection criteria being r^2^, variance cut-off at 0.1 with auto-scaling, the model was built.

### Model validation

Many statistical parameters like n (number of compounds in regression), k (number of variables), degree of freedom, optimum component (number of optimum PLS components in the model), r^2 ^(squared correlation coefficient), F-test (Fischer's value), q^2 ^(cross-validated correlation coefficient), pred_r^2 ^(r^2 ^for external test set), Z score (randomisation test), best_ran_q^2 ^(highest r^2 ^value in the randomisation test) and best_ran_r^2 ^(highest r^2 ^value in the randomisation test) need to be taken into account to consider the model as a robust one. For a model to be statistically significant, the following conditions should be satisfied: r^2^, q^2 ^> 0.6 and pred_r^2 ^> 0.5. Since, F-test gives an idea of the chances of failure of the model, a value greater than 30 is considered to be good. On the other hand, low standard error values establish absolute quality of the model.

### Internal and external validation

For internal validation using leave-one-out method, the cross-validated coefficient, q^2 ^is calculated using the given equation:

$$q^{2}=1-\frac{\sum^{{\left(y_{i}-{\hat{y}}_{i}\right)}^{2}}}{\sum^{{\left(y_{i}-y_{mean}\right)}^{2}}}$$

where $y_{i}$ and ${\hat{y}}_{i}$ are the actual and predicted activities of the  ith (i = 1-26 in Additional File 1) molecule in the training set, respectively, and $y_{mean}$ is the average activity of all the molecules in the training set.

For external validation, the pred_r^2 ^value that gives an account of the statistical correlation between predicted and actual activities of the test set compounds was calculated as follows:

$$pred\text{\_}r^{2}=1-\frac{\sum^{{\left(y_{i}-{\hat{y}}_{i}\right)}^{2}}}{\sum^{{\left(y_{i}-y_{mean}\right)}^{2}}}$$

where $y_{i}$ and ${\hat{y}}_{i}$ are the actual and predicted activities of the  ith molecule (i = 27-36 in Additional File 1) in the test set, respectively, and $y_{mean}$ is the average activity of all the molecules in the training set.

To avoid the risk of chance correlation, Y randomisation test was carried out by comparing the resultant linear model with those derived from random data set [39]. Various models were built on random datasets generated by rearranging the molecules in the training set so as to compare them with the obtained G-QSAR model on the basis of Z-score. A Z-score value is calculated by the following formula:

$$Z\;score=\frac{\left(h-\mu \right)}{\sigma }$$

where  h is the q^2 ^value calculated for the actual data set,  μis the average q^2 ^and  σ is the standard deviation calculated for various models built on different random data sets.

### Combinatorial library generation

A combinatorial library was generated using the *LeadGrow *module of VLife MDS using the template used before and a list of substituent chemical groups to be added to only three substitution sites R4, R5 and R6 based on the calculated descriptors. A library of 4257 compounds was created by substituting electronegative and electropositive atoms, alkyl groups, cyclic groups, aromatic rings and other bulky groups. Their biological activities were predicted using the G-QSAR model obtained.

### Protein modelling and ligand docking

In order to investigate the extent of interactions involved between the most active compounds obtained from the combinatorial library, these compounds were docked to the homology modelled B-chain of tubulin protein using Schrodinger's Glide Module [40-42]. The homology model was obtained using Modeller 9.11 [43-46] taking *Bos taurus *tubulin B-chain [PDB ID: 1SA0] as the template [12]. The resultant homology model was validated using online PSVS suite [47] which comprises a number of software namely, DSSP [48,49], pdbStat (5.9), AutoAssign [50], RPF Analysis [51,52], PDB validation server, Verify3D (1.0) [53], ProsaII (2003) [54], PROCHECK (3.5.4) [55]. Along with these, Molprobity [56] programs and other software are also present.

The tubulin homology model was optimized for docking using Schrodinger's protein preparation wizard [57]. A total of 48 compounds were docked targeting the α-β interfacial site of tubulin heterodimer. Glide works by creating a cubic grid (20 Å side) around the user-specified critical residues and directing the approaching ligand at the specific site. Extra-precision (XP) docking was used to screen compounds with high binding affinity for tubulin. XP docking serves the purpose of correlating good poses with good scores and discarding the false positives [58]. Screening of compounds by docking for potential candidature as therapeutics has been a popular avenue in computational drug design [59-61]. The various interactions involved between the highly active compounds were evaluated using Ligplot [62].

### Molecular Dynamics simulations of the modelled protein and docked complexes

To obtain energetically stable conformation of the modelled protein target and to get an insight into the stability of protein-ligand complexes, molecular dynamic simulations were carried out using Desmond Molecular Dynamics module [63-65] of Schrodinger Maestro by applying optimized Potentials for Liquid Simulations (OPLS) all-atom force field 2005. Prepared protein-ligand complexes were solvated with TI4P water model in a triclinic periodic boundary box for MD simulations. To prevent the direct interaction of protein complex with its own periodic image a boundary box is created and distance between protein complex and box wall is kept at 10 Å. Steepest descent method was used to minimize the energy of the prepared structures for a maximum of 5000 steps till a threshold of 25 kcal/mol/Å is achieved, which was then followed by Low-memory Broyden-Fletcher-Goldfarb-Shanno quasi-Newtonian minimizer until a convergence threshold of 1 kcal/mol/Å was met. Other parameters were kept as default for system equilibration. MD simulations were carried out for 10 ns at a constant temperature of 300 K, pressure 1 atm and at time step of 2 femtoseconds (fs). Long range electrostatic interactions were calculated using smooth particle mesh Ewald method [66] which was occurring during the MD simulations and coulombic short range interaction was calculated using a cut-off scheme, with a cut-off radius of 9 Å for calculation. The protein ligand complex was prepared for MD simulations using the above mentioned parameters. MD Simulation was then carried for a time period of 10 ns.

The root mean square deviation values (RMSD) for two top scoring ligands were calculated for the entire simulations trajectory with reference to their respective first frames. Radius of gyration (ROG) analyses was carried out for all the frames MD simulation of IkB kinase beta and ligand complex.

### Calculation of ADMET properties

The toxicity of final two compounds was evaluated using the Quikprop module [67]of Schrodinger suite which predicts various molecular properties and also provides ranges for comparing these properties with 95% of already available drugs. The analysis was followed by a toxicity analysis using an online webserver, admetSAR [68] which reads the smiles format and results in a number of ADMET values. AdmetSAR is a knowledge based tool comprising of ADMET related properties taken from large literatures which are further used to predict properties of unknown compounds.

## Results and discussion

### G-QSAR model developed for arylthioindole derivatives targeting α-β interfacial site of tubulin

We report a fragment based group QSAR model based on a congeneric series of arylthioindole moiety as the template targeting the α-β interfacial site of tubulin. Colchicine is also known to bind at the same site, though arylthioindoles were found to have much higher activity than colchicine against tubulin polymerization. Inhibition of tubulin polymerization can be a potent deterrent to cell division and hence can be seen as an avenue in developing cancer therapeutics. The tubulin D chain (β unit) structure was modelled based on the close homologous protein belonging to *Bos taurus*.

The G-QSAR model obtained can be represented by the following linear equation:

(1) $$\begin{array}{l} \text{pIC}50=(0.536788\times \text{R}4-\text{H Acceptor Count})+\text{(}-0.412083\times \text{R}6-\text{chi}2)+(-0.538397\times \text{R}5-\text{slogp})\\ +(-0.365145\times \text{R}6-\text{NitrogensCount})+(0.00256203\times \text{R}6-\text{Molecular Weight})+4.97922 \end{array}$$

where pIC50 is the negative logarithm of IC_50 _activity values and was taken as the dependent variable in the model development. A total of 6 substitution sites were marked on the arylthioindole moiety which became the basis of fragmenting the derivatives for model development, of which 3 sites namely, R4, R5 and R6 were taken into account. All 2D molecular descriptors are calculated for each fragment generated. The five molecular descriptors selected on which the model is based are **H Acceptor Count **for the substitution site R4, **slogp **for the site R5 and **chi2**, **NitrogensCount **and **Molecular Weight **for the site R6. The multiplied numerical terms associated with the descriptors are the respective coefficients and the last numerical term is the regression constant. Using the model generated, if the values of these descriptors are known for novel compounds, the biological activity in terms of pIC50 can be determined. Out of these, only R4-H Acceptor Count and R6-Molecular Weight are positively contributing to the biological activity and the rest are negatively contributing. To determine the crucial structural features required for good activity, these descriptors are needed.

R4-H Acceptor Count: This descriptor signifies the number of hydrogen bond acceptor atom and corresponds to the R4 site which was originally substituted by either hydrogen atom or oxymethyl (-OCH3) group in the series taken for model development. As it is a positive contributor, the presence of hydrogen bond acceptor atoms renders high biological activity to the molecule. The percentage of its contribution in enhancing the biological activity was 33.877 %.

R6-chi2: In theoretical terms, this descriptor stands for a retention index of second order which is derived directly from gradient retention times. The retention index of a chemical compound is the normalised retention time which is done to convert these into system independent constants in chromatographic analysis. In chromatography, retention time is the time required for a solute to migrate or elute from the column and this property depends upon the physical properties and behaviour of the molecule. The descriptor was calculated for the substitution site R6 present on the indole core and was mainly substituted by groups like hydrogen atom, halogens like Cl, I, Br, F and other groups like NH2, NO2, CH3, OCH3, OCH2CH3, OCH(CH)_3 _etc. This descriptor contributed negatively to the biological activity to an extent of 23.018 %.

R5-slogp: This descriptor also contributed negatively to the biological activity up to an extent of 17.542% and corresponds to the substitution site R5. Slogp describes the value of log of octanol/water partition coefficient (including implicit hydrogens). It is an atomic property model and calculates logP value from the correct protonation state structure. The R5 site was mainly substituted with hydrogen atoms or oxymethyl (-OCH3) groups.

R6-NitrogensCount: As the name suggests, this descriptor stands for the number of nitrogen atoms present in the compound. This physicochemical descriptor contributes negatively to the activity of arylthioindole derivatives by 15.952%. This suggests that a highly electronegative group, in this case, specifically nitrogen is deterrent for the molecule's activity if present at the R6 position. It should avoid a substitution involving nitrogen atoms.

R6-Molecular Weight: This descriptor signifies molecular weight of the compound and is positively contributing in the biological activities by 9.611%. This indicates the importance of substituents with higher molecular weight present at the R6 substitution site.

### Model evaluation and validation of G-QSAR model developed for arylthioindole derived compounds

#### Unicolumn statistics for the chosen training and test set

For model evaluation and validation the complete dataset is divided into training set and test set. The developed model is validated by predicting the inhibitory activity (in terms of pIC_50_) of the training set (known as internal validation) and the test set (known as external validation). The test set chosen can always be evaluated beforehand using the unicolumn statistics (Table 1). The unicolumn statistics can be interpreted in terms of the maximum and minimum of training and test set. The min of test set should be equal or more than the min of training set and the max of the test set should be equal or less than the max of training set. This data is in absolute compliance with the conditions mentioned and shows that the test set is interpolative (derived within the min-max range of the training set). The relative difference of mean and point density distribution (along mean) of the two sets can be derived from the average and standard deviation. In this case, as the average value in the test set is slightly higher than the training set, the presence of relatively more active molecules as compared to the inactive ones is indicated. Also, a higher standard deviation for the training set indicates that training set constitutes widely distributed activity of the molecules as compared to the test set.

**Table 1:** Unicolumn statistics for the training and test set compounds.

	Average	Max	Min	Standard Deviation	Sum
**Training**	5.2573	5.7900	4.7200	0.3389	136.69
**Test**	5.5700	5.6900	5.3800	0.0968	55.77

#### Validation of the final G-QSAR model

The G-QSAR model is evaluated on the basis of certain statistical parameters for both internal and external validation. The number of compounds in the training set was specified by N which is 26. Considering the correlation coefficient, r^2 ^(0.8512), cross-validated correlation coefficient q^2 ^(0.7175), pred_r^2 ^(0.8968), low standard error value, r^2^_se (0.1363), q^2^_se (0.1878) and pred_r^2^_se (0.1127), the model can be stated to be a robust one. Along with this, a high F-test value (65.7699) implied that the model is 99.999 % statistically valid with less than 1 in 10000 chance of failure.

Also with this, the randomization test shows confidence of 100% (Alpha Rand r^2^=0.00000) that the generated model is not random and hence is chosen as the G-QSAR model. Other important statistical parameters have been determined and Z-score is highlighted to emphasize its importance in QSAR model validation (Table 2). The values of selected descriptors for each compound in the dataset have been provided (Additional file 2). The Z score gives an idea about how far away is the observed value from the mean. A Zscore_r^2 ^of 6.48008, Zscore_q^2 ^of 4.73992 and Zscore_pred_r^2 ^of 1.70900 statistically validates the significance of the obtained G-QSAR model. In order to get a better idea of the validity of the model, p values were determined for each correlation coefficient using the corresponding Z scores. The P value_r2 and P value_q2 of less than 0.0001 tells that the hypothesis is statistically significant. The P value_pred_r2 of 0.0875 is although, not quite statistically significant.

**Table 2:** The statistical parameters calculated for developed G-QSAR model.

Dep Variable	ZScore r^2^	ZScore q^2^	Best Rand r^2^	Best Rand q^2^	Alpha Rand r^2^	Alpha Rand q^2^	Z Score Pred r^2^	best Rand Pred r^2^	alpha Rand Pred r^2^
**pIC50**	6.4800	4.7399	0.3433	0.3414	0.00000	0.00003	1.7090	0.5865	0.05000

**Figure 1:**
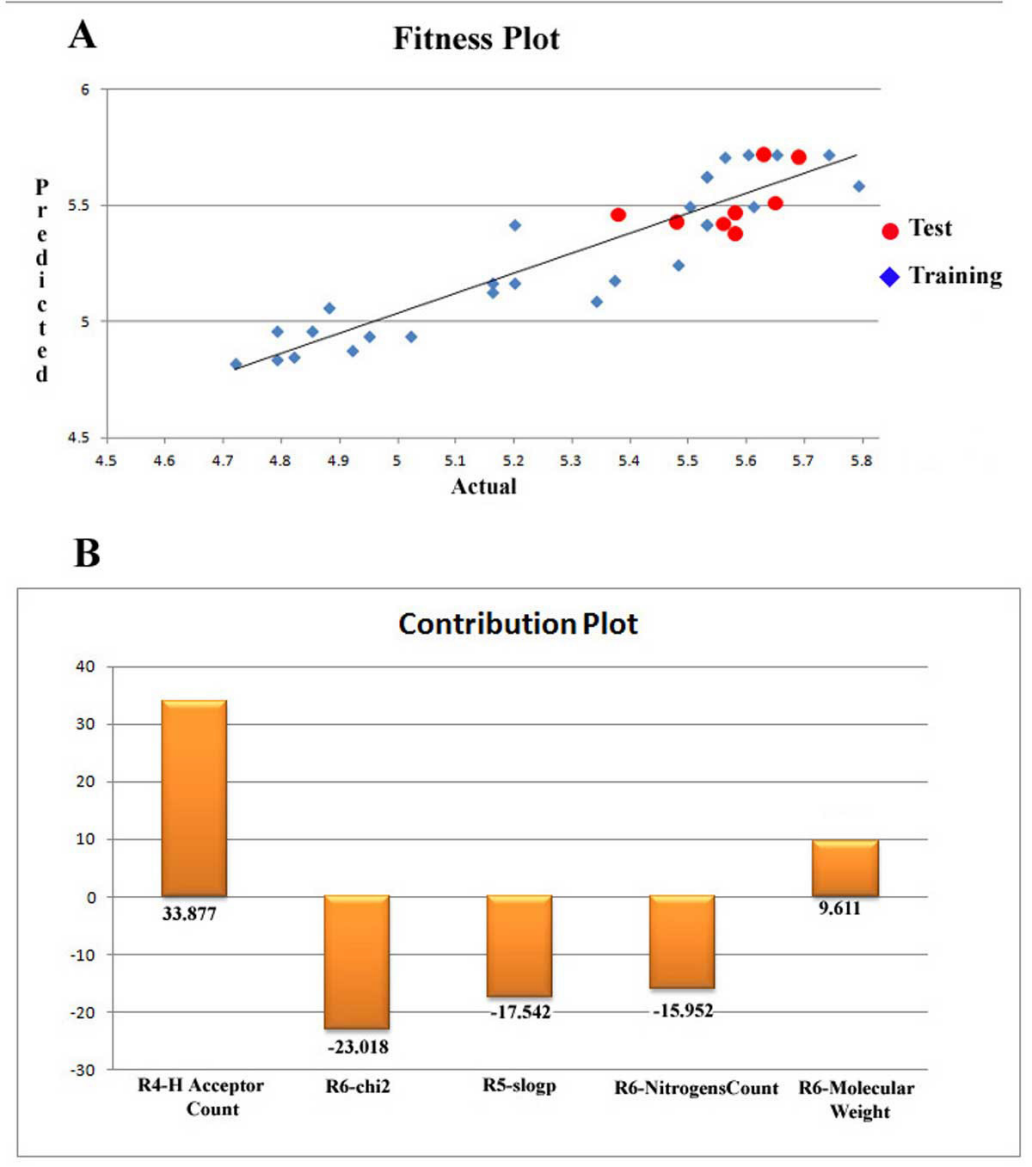
(a) Fitness Plot of the G-QSAR model obtained for actual and predicted activities. (b) Contribution Plot of the selected descriptors R4-H Acceptor Count, R6-chi2, R5-slogp, R6-NitrogensCount and R6-Mol.Wt.

**Figure 2:**
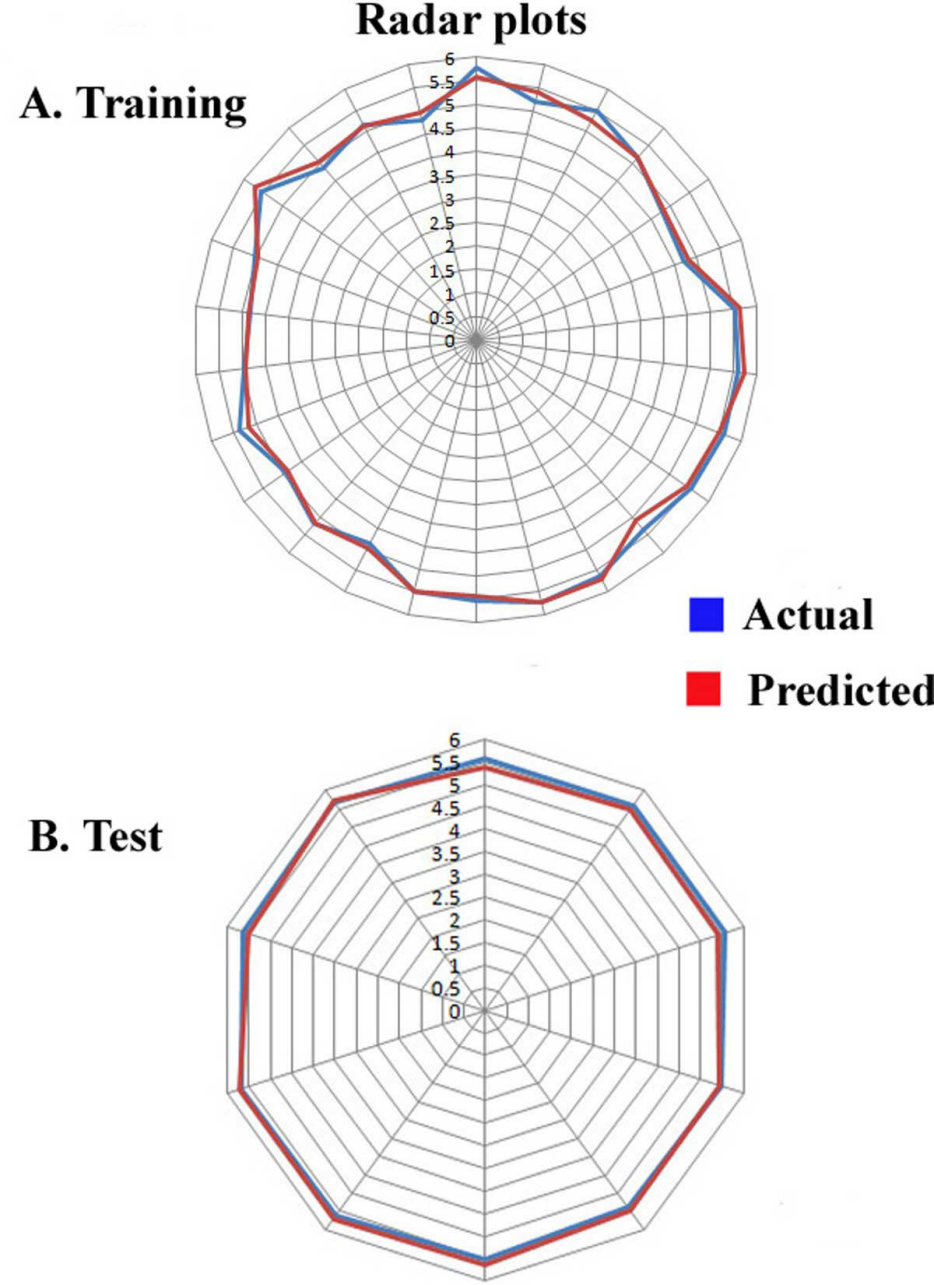
Radar graphs plotting the predicted and actual activities for (a) Training set and (b) Test set.

The robustness of the model is better understood through the linear graphical representation between actual and predicted activities of the 36 compounds along with the contribution plot for each descriptor (Figure 1). The linear graphical representation shows the extent of variation between the actual and predicted activities of the congeneric set. The larger the distance of training and test set points from the regression line, more is the difference between the actual and the predicted activity values. The contribution of each descriptor specifies the properties that should be present in the drug lead for enhancing its inhibitory activity. Presence of descriptors with positive contribution increases its inhibitory activity while descriptors with negative contribution decrease the same. Radar plots for training and test sets are given (Figure 2). The radar graphs depict the difference in the actual and predicted activities for the training and the test sets separately by the extent of overlap between blue (actual activity) and red (predicted activity) lines. The radar plot for training set represents a good r^2 ^value if the two lines show a good overlap while for the test set a good overlap represents high pred_r^2 ^value.

### Activity prediction of combinatorial library generated using arylthioindole moiety as template

A combinatorial library was constructed resulting in 4257 compounds after substituting the R4, R5 and R6 sites with various chemical groups for which the descriptors were calculated. The three sites R4, R5 and R6 were populated by single atoms like N, C, O and halogens like Cl, F, Br and I, by alkyl groups and aromatic rings like phenoxy, phenyl, furan, pyrrole, pyridine, imidazole, 2-thiophene etc along with alkenes, acids, aromatic rings, aliphatic rings and other groups such as -O-CH3, -O-C2H5, amide, cyanide, cyanate, isocyanate, -C=N, -N=C, azo, hydrazo, benz etc were also added. The biological activities of these compounds were predicted using the G-QSAR model. 48 arylthioindole derivatives were further selected to obtain mechanistic insights into their inhibitory properties based on high predicted activity scores and extrapolation values. Ideally, the compounds with extrapolation values being zero or close to zero are considered good for further analysis. The compound with the highest predicted activity score of 6.047, temp0592 was substituted with an electronegative H-bond acceptor NH2 group at the R4 site, a hydrogen atom at the R5 site and a sulphur atom at the R6 site which has a molecular weight of 32.065. It is heavier than CH2OH having a molecular weight of 31.021 which was substituted at the same site in temp0598 and has lower activity value of 6.042 as R6-Molecular Weight is a positively contributing descriptor. Similarly, temp0596 goes further down on activity value as R6 site is substituted by the ethyl group and hence having lesser molecular weight. The presence of H bond Acceptor at the R4 site enhances the inhibitory activity of arylthioindole derivatives as in temp0592, temp1656, temp2454 and many more. All these observations can be summed up as the presence of an electronegative H bond acceptor at the R4 site and a high molecular weight group at the R6 site enhances the activity of arylthioindoles. But an increased slogp value at the R5 site, increased retention index chi2 and nitrogen atom count at the R6 site decreases the biological activity. These observations establish the importance of such substituents at these sites to enhance the tubulin inhibitory activity of arylthioindole derivatives. The predicted activity values with respect to the three substitution sites R4, R5 and R6 have been provided for 20 top scoring compounds (Table 3).

**Table 3:** Predicted activity values of the combinatorial library with respect to R4, R5 and R6 site substitutions.

**S. No**.	Compound	R4	R5	R6	Predicted activity
1	temp0592	N	O	S	6.047
2	temp1656	OH	O	S	6.047
3	temp2454	phenoxy	O	S	6.047
4	temp3518	amine	O	S	6.047
5	temp1655	OH	O	P	6.047
6	temp2453	phenoxy	O	P	6.047
7	temp3783	-O-CH3	O	P	6.047
8	temp0605	N	O	-O-CH3	6.042
9	temp1662	OH	O	OH	6.042
10	temp2467	phenoxy	O	-O-CH3	6.042
11	temp3531	amine	O	-O-CH3	6.042
12	temp3790	-O-CH3	O	OH	6.042
13	temp4056	-O-C2H5	O	OH	6.042
14	temp4063	-O-C2H5	O	-O-CH3	6.042
15	temp3788	-O-CH3	O	ethyl	6.037
16	temp4054	-O-C2H5	O	ethyl	6.037
17	temp3521	amine	O	F	6.011
18	temp3787	-O-CH3	O	F	6.011
19	temp4052	-O-C2H5	O	O	6.006
20	temp3789	-O-CH3	O	Methyl	6.001

### Evaluation of screened compounds docked against α-β interfacial site of tubulin heterodimer

#### 1. Validation of homology based model of human tubulin

**Figure 3:**
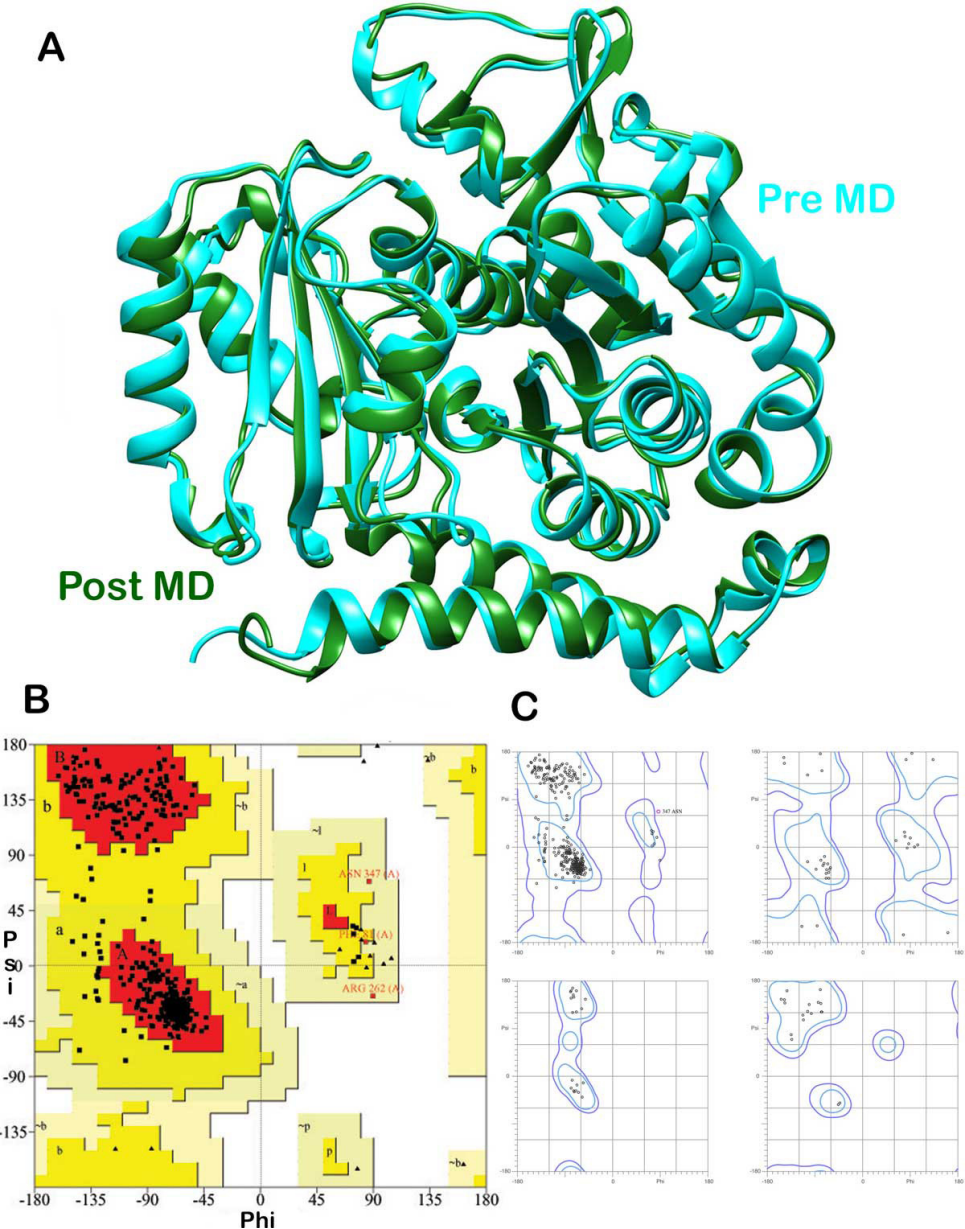
(a) Diagrammatic representation of structural differences between the pre MD and post MD simulated homology based model of human tubulin. (b) Ramchandran plot analysis from PSVS PROCHECK module. (c) Ramchandran plot analysis from Richardson lab's Molprobity module for glycine, proline and pre-proline separately and rest of the residues.

The homology based protein structure of human tubulin was validated using PSVS validation suite. The conformational shifts in the protein model encountered after molecular dynamic simulations has been depicted (Figure 3A). After obtaining the MD simulated structure, Ramchandran plot analysis was carried out to find the percentage of amino acid residues falling in the sterically allowed and disallowed regions. According to PROCHECK analysis, 90.4 % residues fall under the most favoured regions and no residues in the disallowed regions. Of the 431 residues, 34 were glycine and 20 were proline and were analysed separately. A model having over 90% residues falling in the favoured region can be considered to be good one on account of the analysis of 118 structures of resolution 2.0 Å. Thus, the human tubulin model is a good one and can be considered for further analysis. The Ramchandran plot analysis using PROCHECK has been depicted (Figure 3B). According Richardson's Lab molprobity analysis, 97 % of the residues fall under the favoured regions and 0.2 % under the disallowed regions. Just one outlier ASN347 was encountered. As Molprobity analyses Glycine, Proline and pre-proline separately owing to basic physicochemical differences in comparison to other amino acids, 4 different Ramchandran plots are given (Figure 3C). The average PROCHECK G factor value for phi-psi angles was -0.15 and for all dihedral angles was -0.23. The G score is the log odds score of observed distribution of torsion angles and covalent geometries and quantifies the goodness of the structure. The high the value of G score, better is that dihedral angle in the structure in terms of falling under the favourable regions in a Ramchandran plot. As the average G score is negative, high number of residues was seen having distortions in dihedral angles.

#### 2. Screening of combinatorial library through docking and evaluation of the top scoring compounds

**Figure 4:**
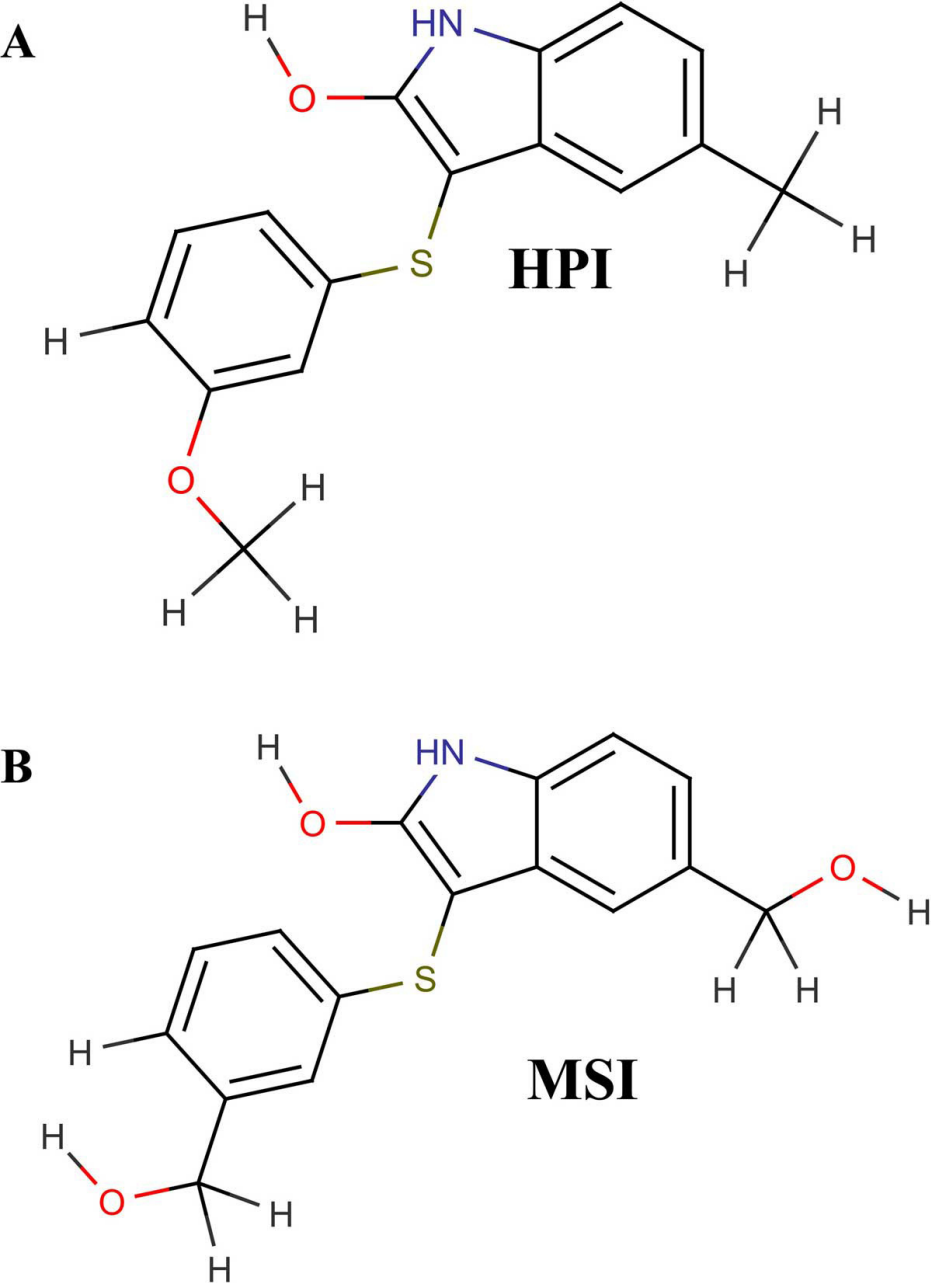
The structures of the two reported compounds (a) HPI and (b) MSI.

**Figure 5:**
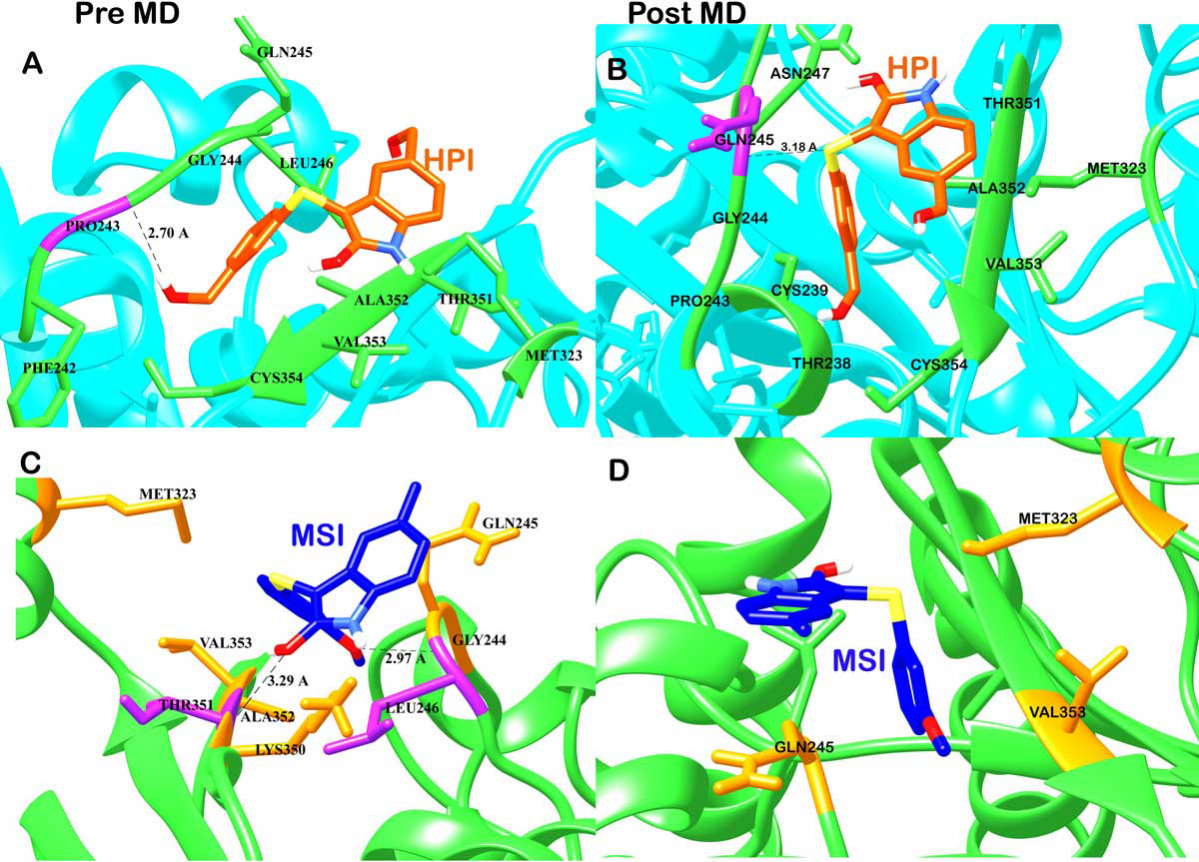
Diagrammatic representation of residues involved in various interactions with (a) HPI (shown in orange) forming hydrogen bond with Pro243 (purple) and residues involved in hydrophobic interactions (shown in green) (b) The post MD interactions of HPI showing one H bond with Gln245. (c) MSI (shown in blue) forming hydrogen bonds with Gly244 and Thr351 (purple) and residues involved in hydrophobic interactions are shown in orange. (d) The post MD interaction of MSI having no hydrogen bonds.

The 48 compounds having high predicted activity values chosen from the combinatorial library were docked against the tubulin assembly targeting the α-β interfacial site of the homology based protein structure of tubulin chain B. The top two compounds showing maximum affinity for the colchicine binding site were selected. Both the target-ligand complexes obtained were MD simulated and their interactions observed. The first compound with high affinity for tubulin, temp1662 named *5-(hydroxymethyl)-3-{[3-(hydroxymethyl) phenyl] sulfanyl}-H-indol-2-ol ***(HPI) **(Figure 4A) was involved in both hydrogen and hydrophobic interactions. The O atom of Pro243 formed a 2.72 Å long hydrogen atom with O1 atom of HPI and the rest others like Phe242, Gly244, Gln245, Leu246, Met323, Ala352, Val353, Cys354 and Thr351 were found to be involved in hydrophobic interactions (Figure 5A). After performing MD simulations in two slots of 10ns each, the stable average structure was obtained from 10 to 20ns. New interaction patterns could be seen post simulation with a 3.18 Å long H bond formed between the S atom of HPI and N atom of Gln245 which was involved in hydrophobic interaction before MD simulations. HPI seemed to have shifted from the earlier position owing to new hydrophobic interactions being formed including, Asn247, Pro243, Thr238 and Cys239. Rest all interactions were conserved with Leu246, gly244, Met323, Thr351, Ala352, Val353 and Cys354 (Figure 5B).

**Figure 6:**
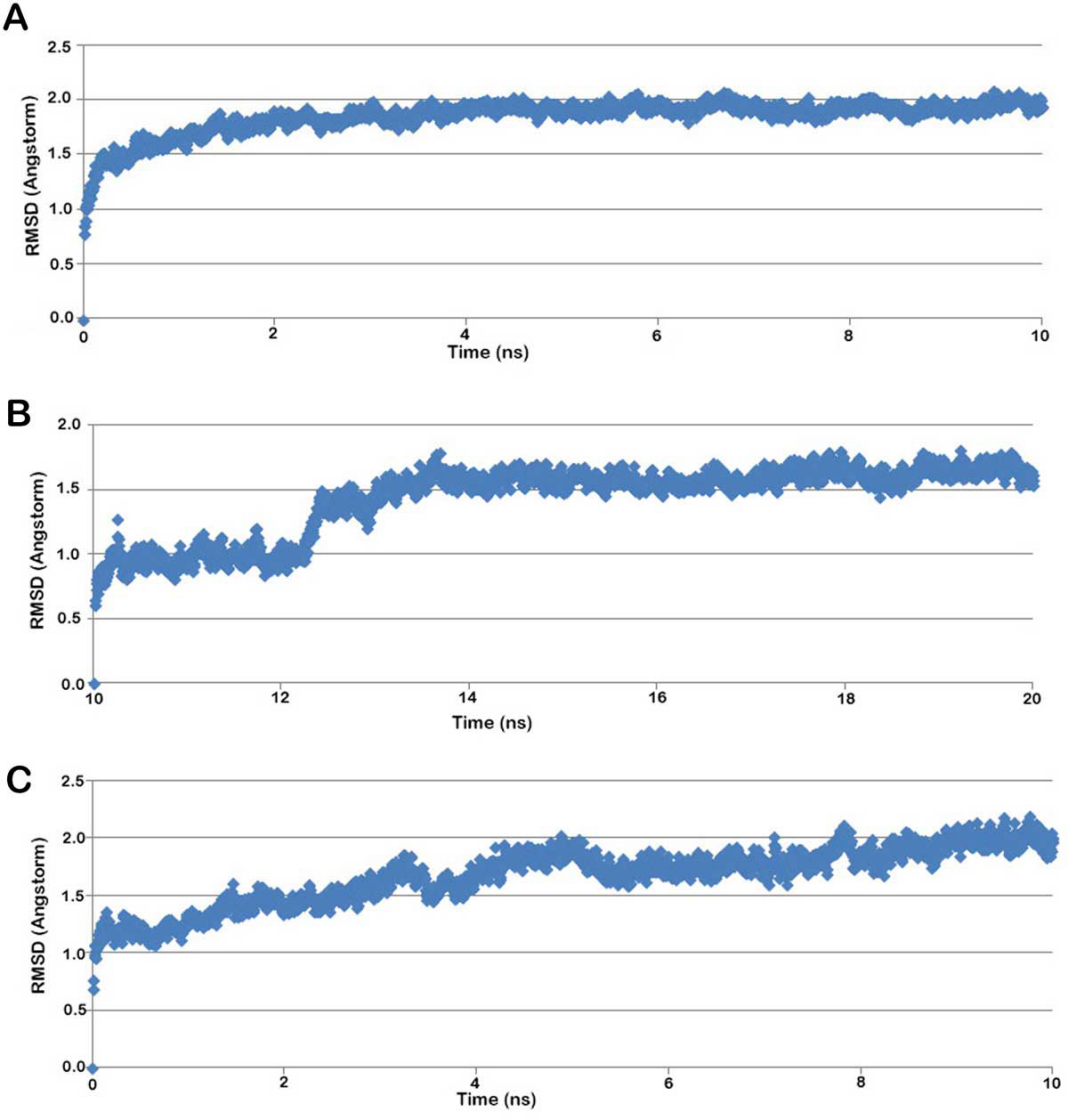
The graphical depiction of RMSD trajectory of (a) homology based tubulin model (from 0-10 ns) (b) target-HPI complex (from 10-20 ns) and (c) target-MSI complex (from 0-10 ns).

**Figure 7:**
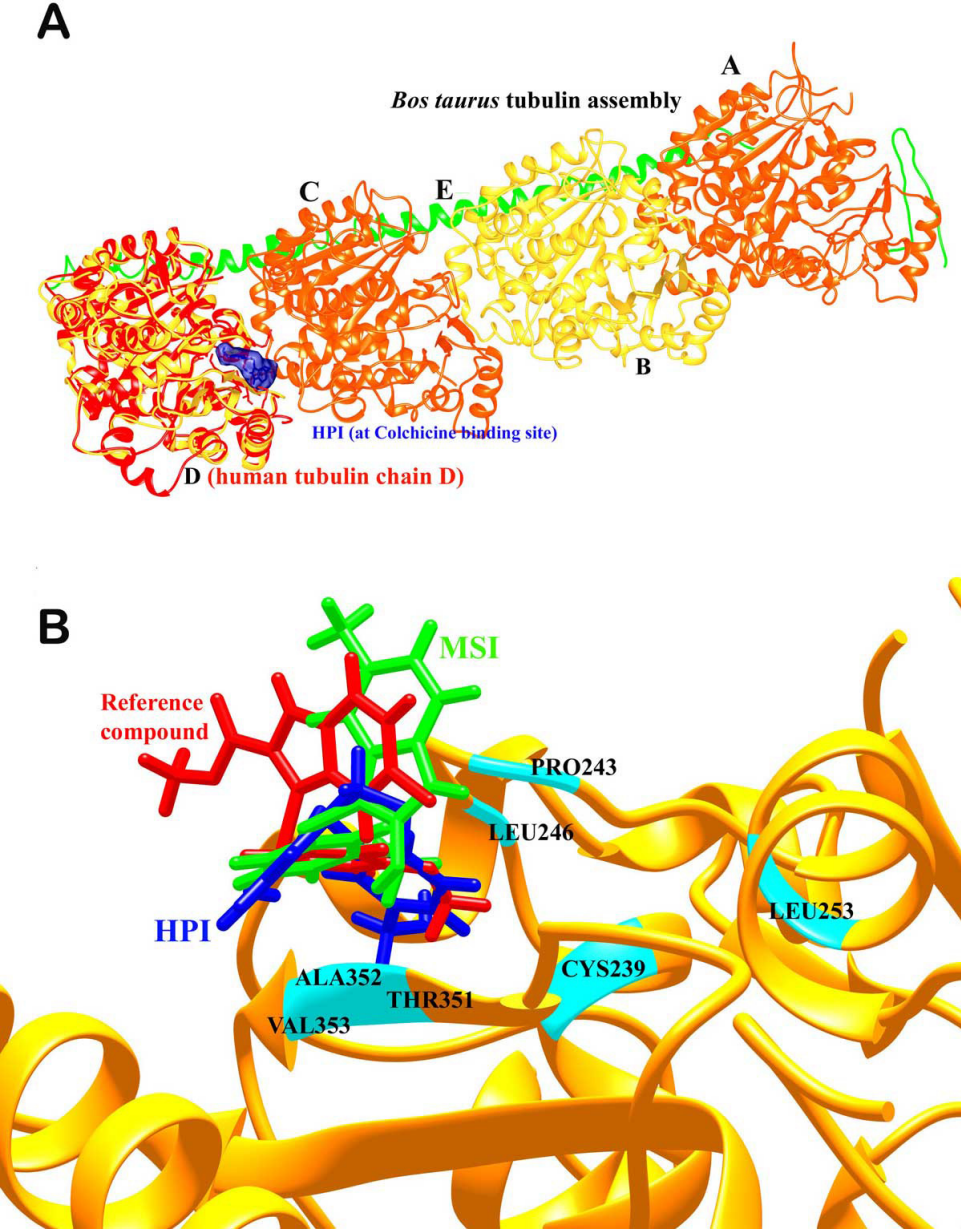
(a) Depiction of the complete *Bos taurus *tubulin assembly comprising chains A, B, C, D and E of which A and C belong to the α chain (shown in orange); B and D belong to the β chain (shown in yellow). The modelled human tubulin β-chain has been compared (in red) and the ligand is shown in blue bound at the α-β interfacial colchicine binding site. (b) Depiction of the binding modes of HPI (in blue) and MSI (in green) compared to the most active arylthioindole derivative (in red) taken as the reference compound and important residues (in cyan).

The second compound temp3789 named *3-[(3-methoxyphenyl) sulfanyl]-5-methyl-1H-indol-2-ol ***(MSI) **(Figure 4B) was also found to be involved in both hydrogen bonds as well as hydrophobic interactions with the colchicine binding site in the modelled human tubulin protein. Leu246 and Thr351 were involved in forming hydrogen bonds of distances 2.97 and 3.29 Å respectively with MSI and Gly244, Gln245, Met323, Lys350, Ala352 and Val353 were involved in hydrophobic bonding. The hydrogen bonds were formed between Nitrogen atom of Lys246 with the O1 atom and Nitrogen atom of Thr351 with O2 atom of MSI (Figure 5C). After MD simulations of 10 ns, the interactions were seen to be reduced to hydrophobic interaction with Met323, Gln245 and Val353 (Figure 5D). The RMSD graphs for depicting the course of three MD simulations i.e. tubulin homology model (Figure 6A), ligand complexes with HPI (Figure 6B) and MSI (Figure 6C) have been given. When compared to the interactions involved between the most active arylthioindole derivative and tubulin, similar mode of action was discovered forming a 2.86Å long H-bond between N atom of Lys246 with O1 atom of the reference compound. Hydrophobic interactions were found to exist involving a few but same residues as those involved with HPI and MSI including Gly244, Gln245, Lys350, Ala352 and Val353 (Table 4). Thus, the two reported compounds HPI and MSI bind to the same colchicine binding site at the α-β interfacial cavity of the tubulin assembly and show good binding affinity (Figure 7). The predicted activities of HPI and MSI were 6.042 and 6.001 based on the G-QSAR model and thus, are in absolute concurrence with the docking results in terms of high binding affinity with the tubulin assembly having similar mode of action as that of colchicine but with much higher biological activity than the most active compound of the congeneric series taken for this study.

**Table 4:** Tubulin residues that are involved in hydrogen and hydrophobic interactions with HPI and MSI respectively in comparison with the reference compound.

	Hydrogen bonding	Hydrophobic interactions
Most active arylthioindole derivative (reference)	Leu246	Gly244, Lys350, Ala352, Val353

HPI	Pro243	Phe242, Gly244, Gln245, Leu246, Ala352, Val353, Cys354, Met323, Thr351

MSI	Leu246, Thr351	Met323, Lys350, Gln245, Gly244, Ala352, Val353

### ADMET analysis of the two top compounds, HPI & MSI

A total of 50 ADME properties were calculated using the Quikprop module. The first property #star depicts the number of properties that fall out of the similarity criteria of 95% of known drugs. The values for HPI and MSI were 0 and 1 respectively and signify that no molecular property of HPI falls out of the similarity criteria while only one criterion falls out for MSI. Another property CNS (central nervous system activity) ranging from -2 (inactive) to 2 (active) resulted in a value of -2 for HPI and zero for MSI. QPlogBB is the predicted brain/blood partition coefficient which had a value of -1.416 and -0.218 for HPI and MSI respectively. Descriptors including SASA (solvent accessible surface area), its hydrophobic component FOSA (saturated carbon and attached hydrogen) and hydrophilic component FISA (N, O and H on heteroatoms) were also predicted within the recommended ranges for both compounds. The compound HPI had a value of 81.27% and MSI had a value of 100% for percent human oral absorption descriptor. The predicted skin permeability factor QPlogkp resulted in a value of -3.342 and -1.635 for the two compounds HPI and MSI respectively. Another set of descriptors including QPlogPC16 (Free energy of solvation in hexadecane), QPlogPoct (Free energy of solvation in octanol), QPlogPw (Free energy of solvation in water) give the distribution of compounds in the body. QPlogPo/w (Predicted octanol/water partition coefficient) is the partition coefficient that gives an idea of the hydrophobic nature of the chemical compounds and resulted in high values of 2.244 and 4.197 for MSI and HPI respectively suggesting their easy absorption through the lipid bilayer. It also finds out the number of various chemical groups present in the test compounds for e.g. amide, acid etc. The ionization potential and electron affinities (in electron Volts) for both the compounds were also obtained being 8.489 and 0.349 for HPI and 8.504 and 0.392 for MSI respectively. Lastly, it also evaluated the two compounds on the basis of Lipinski Rule-of-five and Jorgensen Rule-of-three violations which resulted in zero violations for both, HPI and MSI.

Certain other ADMET properties calculated by the online server admetSAR have also been evaluated. The absorption factors for HPI can be summarised in terms of blood brain barrier (BBB), human intestinal absorption (HIA) and CaCo2 permeability and resulted in a positive value (value above the prescribed threshold suggesting good permeability) with high probabilities. A compound with >30% HIA is absorbed easily. Similarly, CaCo2 permeability is taken as the *in vitro *model of human small intestinal mucosa as it provides a physical and biochemical barrier to ions and small molecules and hence must be tested for orally administered drugs. Under the metabolism category, HPI was found to be a non-substrate for CYP450 2C9 and 2D6, substrate for CYP450 3A4, inhibitor for 1A2 and 2C9 and non-inhibitor for 2C19 and 3A4 and thus, having a high cytochrome inhibitory promiscuity. Under the toxicity category, it was found to be non-carcinogenic but toxic for Fish, *Tetrahymena *and Honey Bee.

Similarly, MSI was also evaluated which resulted in positive BBB and HIA permeability but no CaCo2 permeability. Under the metabolism category, it was found to be a non-substrate for CYP450 2C9, 2D6 and 3A4, an inhibitor of 1A2, 2C9 and 2C19 and non-inhibitor of CYP450 2D6 and 3A4 and thus, showed high cytochrome inhibitory promiscuity like HPI. Under the toxicity category, it was found to be non toxic for AMES test thus a non-carcinogen while having high toxicity for fish and honey bee but no toxicity for *Tetrahymena*. Thus, MSI can be selected for further evaluation as the drug candidate having low toxicity values as predicted.

## Conclusions

In this study, we reported a novel fragment-based group QSAR approach exploiting the disintegration of tubulin assembly leading to interruption in cell division and eventually apoptosis as a potent avenue in cancer management. Tubulin heterodimers when assembled form the major building blocks of microtubules and hence have important functional role in maintaining cell structural integrity, motility and division in terms of spindle fibre formation. Inhibition of the tubulin formation is seen as a viable option for deterring the growth of cancerous cells. The arylthioindole moiety has been considered to be a potential anti-tubulin compound having similar mode of action as other agents involved in cell division arrest like colchicine and combretastatin. A G-QSAR model can provide significant results in terms of the crucial chemical fragments required for enhancing the activity of an already present template compound known to be inhibitory to our target of interest. A statistically robust G-QSAR model with the test set comprising 10 compounds out of a total of 36 compounds gave an insight into the contribution of various substitutions at the three sites R4, R5 and R6 for which 5 descriptors were calculated namely, R4-H AcceptorCount, R6-chi2, R5-slogp, R6-NitrogensCount, R6-Molecular Weight. A combinatorial library was created by varying substituents at these sites and their activity was predicted based on the model. All compounds having an electronegative H-bond acceptor at the R4 site and a substituent with high molecular weight at the R6 site were found to have high activities (pIC50) while the presence of nitrogen atom at R6 site resulted in decrease of biological activity of the arylthioindole derivatives. All highly active compounds were docked at the colchicine binding α-β interfacial site of the homology-based human tubulin chain B. After final analysis of the binding affinities and interactions involved, we reported two compounds, HPI and MSI with predicted activities of 6.042 and 6.001respectively in terms of pIC50. Out of the two, the second compound MSI promises to be the first choice for further evaluation as the drug candidate, being less toxic in comparison to HPI. The present study elucidated the crucial structural requirements to enhance the anti-tubulin activity of arylthioindole derivatives. The study provides a better understanding of the inhibitory roles of the identified compounds in microtubule disassembly and paves way for consideration of these compounds as potent anticancer leads.

## Competing interests

The authors declare that they have no competing interests.

## Authors' contributions

CT and AGr designed the methods and experimental setup. CT and AG carried out the implementation of the various methods and were assisted by AG and SG. CT and AGr wrote the manuscript. All authors have read and approved the final manuscript.

## Supplementary Material

Additional file 1 Table S1 - Structures and anti-cancer activity values of arylthioindole derivatives used in this study. Click here for file

Additional file 2 Table S2 - Calculated descriptor values and predicted activity of arylthioindole derivatives. Click here for file

## Declaration

AG would like to acknowledge financial support from Department of Science and Technology, Government of India towards publication of this article.

This article has been published as part of *BMC Genomics *Volume 15 Supplement 9, 2014: Thirteenth International Conference on Bioinformatics (InCoB2014): Computational Biology. The full contents of the supplement are available online at http://www.biomedcentral.com/bmcgenomics/supplements/15/S9.

## References

[B1] Checchi PM Nettles JH Zhou J Snyder JP Joshi HC Microtubule-interacting drugs for cancer treatment Trends Pharmacol Sci 2003 24 7 361 365 12871669 10.1016/S0165-6147(03)00161-5

[B2] Nicolaou K Vourloumis D Li T Pastor J Winssinger N He Y Ninkovic S Sarabia F Vallberg H Roschangar F Designed epothilones: Combinatorial synthesis, tubulin assembly properties, abd cytotoxic action against taxol-resistant tumor ells Angew Chem Int Edit 1997 36 19 2097 2103

[B3] Pettit GR Singh SB Niven ML Hamel E Schmidt JM Isolation, structure, and synthesis of combretastatins A-1 and B-1, potent new inhibitors of microtubule assembly, derived from Combretum caffrum J Nat Prod 1987 50 1 119 131 3598594 10.1021/np50049a016

[B4] Mooberry SL Microtubules as a target for anticancer drugs New Frontiers and Treatment Paradigms for Metastatic Breast Cancer 2011 28 7

[B5] Lockwood AH Tubulin assembly protein: immunochemical and immunofluorescent studies on its function and distribution in microtubules and cultured cells Cell 1978 13 4 613 627 350414 10.1016/0092-8674(78)90212-x

[B6] Nogales E Structural insights into microtubule function Annu Rev Biochem 2000 69 1 277 302 10966460 10.1146/annurev.biochem.69.1.277

[B7] Hammond JW Cai D Verhey KJ Tubulin modifications and their cellular functions Curr Opin Cell Biol 2008 20 1 71 76 18226514 10.1016/j.ceb.2007.11.010 PMC2274889

[B8] Pellegrini F Budman DR Review: tubulin function, action of antitubulin drugs, and new drug development Cancer Invest 2005 23 3 264 273 15948296 10.1081/cnv-200055970

[B9] Prinz H Ishii Y Hirano T Stoiber T Camacho Gomez JA Schmidt P Düssmann H Burger AM Prehn JH Günther EG Novel benzylidene-9 (10 H)-anthracenones as highly active antimicrotubule agents. Synthesis, antiproliferative activity, and inhibition of tubulin polymerization J Med Chem 2003 46 15 3382 3394 12852768 10.1021/jm0307685

[B10] Prakasham A Saxena A Luqman S Chanda D Kaur T Gupta A Yadav D Chanotiya C Shanker K Khan F Synthesis and anticancer activity of 2-benzylidene indanones through inhibiting tubulin polymerization Bioorgan Med Chem 2012 20 9 3049 3057 10.1016/j.bmc.2012.02.057 22472045

[B11] Wilson L Meza I The mechanism of action of colchicine. Colchicine binding properties of sea urchin sperm tail outer doublet tubulin J Cell Biol 1973 58 3 709 719 4747924 10.1083/jcb.58.3.709 PMC2109066

[B12] Ravelli RB Gigant B Curmi PA Jourdain I Lachkar S Sobel A Knossow M Insight into tubulin regulation from a complex with colchicine and a stathmin-like domain Nature 2004 428 6979 198 202 15014504 10.1038/nature02393

[B13] Jordan MA Thrower D Wilson L Effects of vinblastine, podophyllotoxin and nocodazole on mitotic spindles. Implications for the role of microtubule dynamics in mitosis J cell Sci 1992 102 3 401 416 1506423 10.1242/jcs.102.3.401

[B14] Lin CM Ho HH Pettit GR Hamel E Antimitotic natural products combretastatin A-4 and combretastatin A-2: studies on the mechanism of their inhibition of the binding of colchicine to tubulin Biochemistry 1989 28 17 6984 6991 2819042 10.1021/bi00443a031

[B15] Shan YS Zhang J Liu Z Wang M Dong Y Developments of combretastatin A-4 derivatives as anticancer agents Curr Med Chem 2011 18 4 523 538 21143124 10.2174/092986711794480221

[B16] Risinger AL Mooberry SL Microtubules as a target in cancer therapy Cytoskeleton and Human Disease 2012 Springer 203 221

[B17] Beckers T Mahboobi S Natural, semisynthetic and synthetic microtubule inhibitors for cancer therapy Drugs Future 2003 28 767 785

[B18] Mani S Macapinlac M Jr Goel S Verdier-Pinard D Fojo T Rothenberg M Colevas D The clinical development of new mitotic inhibitors that stabilize the microtubule Anti-cancer Drug 2004 15 6 553 558 10.1097/01.cad.0000131681.21637.b2 15205596

[B19] De Martino G La Regina G Coluccia A Edler MC Barbera MC Brancale A Wilcox E Hamel E Artico M Silvestri R Arylthioindoles, potent inhibitors of tubulin polymerization J Med Chem 2004 47 25 6120 6123 15566282 10.1021/jm049360d

[B20] De Martino G Edler MC La Regina G Coluccia A Barbera MC Barrow D Nicholson RI Chiosis G Brancale A Hamel E New arylthioindoles: potent inhibitors of tubulin polymerization. 2. Structure-activity relationships and molecular modeling studies J Med Chem 2006 49 3 947 954 16451061 10.1021/jm050809s

[B21] Coluccia A Sabbadin D Brancale A Molecular modelling studies on arylthioindoles as potent inhibitors of tubulin polymerization Eur J Med Chem 2011 46 8 3519 3525 21621885 10.1016/j.ejmech.2011.05.020

[B22] Lu Y Chen J Xiao M Li W Miller DD An overview of tubulin inhibitors that interact with the colchicine binding site Pharm Res 2012 29 11 2943 2971 22814904 10.1007/s11095-012-0828-z PMC3667160

[B23] Massarotti A Coluccia A Silvestri R Sorba G Brancale A The tubulin colchicine domain: a molecular modeling perspective Chem Med Chem 2012 7 1 33 42 21990124 10.1002/cmdc.201100361

[B24] Nogales E Wolf SG Downing KH Structure of the αβ tubulin dimer by electron crystallography Nature 1998 391 6663 199 203 9428769 10.1038/34465

[B25] Liao Sy Miao Tf Chen Jc Lu Hl Zheng Kc Molecular modeling and design of arylthioindole derivatives as tubulin inhibitors Chin J Chem Phy 2009 22 5 473 480

[B26] Brancale A Silvestri R Indole, a core nucleus for potent inhibitors of tubulin polymerization Med Res Rev 2007 27 2 209 238 16788980 10.1002/med.20080

[B27] Goyal M Dhanjal JK Goyal S Tyagi C Hamid R Grover A Development of dual inhibitors against alzheimer's disease using fragment-based QSAR and molecular docking BioMed Res Int 2014 2014 12 10.1155/2014/979606 PMC4075005 25019089

[B28] Ajmani S Jadhav K Kulkarni SA Group-Based QSAR (G-QSAR): Mitigating interpretation challenges in QSAR QSAR Comb Sci 2009 28 1 36 51

[B29] Goodarzi M da Cunha EF Freitas MP Ramalho TC QSAR and docking studies of novel antileishmanial diaryl sulfides and sulfonamides Eur J Med Chem 2010 45 11 4879 4889 20728249 10.1016/j.ejmech.2010.07.060

[B30] Goyal S Dhanjal JK Tyagi C Goyal M Grover A Novel fragment-based QSAR modeling and combinatorial design of pyrazole-derived CRK3 inhibitors as potent antileishmanials Chem Biol Drug Des 2014 84 1 54 62 24447365 10.1111/cbdd.12290

[B31] Goyal S Grover S Dhanjal JK Tyagi C Goyal M Grover A Group-based QSAR and molecular dynamics mechanistic analysis revealing the mode of action of novel piperidinone derived protein-protein inhibitors of p53-MDM2 J Mol Graph Model 2014 51 64 72 24858256 10.1016/j.jmgm.2014.04.015

[B32] Tyagi C Grover S Dhanjal J Goyal S Goyal M Grover A Mechanistic insights into mode of action of novel natural cathepsin L inhibitors BMC Genomics 2013 14 Suppl 8 S10 24564425 10.1186/1471-2164-14-S8-S10 PMC4042235

[B33] Ajmani S Kulkarni SA Application of GQSAR for scaffold hopping and lead optimization in multitarget inhibitors Mol Inform 2012 31 6-7 473 490 10.1002/minf.201100160 27477466

[B34] Giansanti V Piscitelli F Camboni T Prosperi E La Regina G Parks M Silvestri R Scovassi AI Arylthioindoles: Promising compounds against cancer cell proliferation Oncol Lett 2010 1 1 109 112 22966266 10.3892/ol_00000020 PMC3436460

[B35] La Regina G Edler MC Brancale A Kandil S Coluccia A Piscitelli F Hamel E De Martino G Matesanz R Díaz JF Arylthioindole inhibitors of tubulin polymerization. 3. Biological evaluation, structure-activity relationships and molecular modeling studies J Med Chem 2007 50 12 2865 2874 17497841 10.1021/jm061479u

[B36] MarvinSketch 2013 v. 5.12.1, ChemAxon http://www.chemaxon.com

[B37] VLifeMDS: Molecular Design Suite 2010 v. 4.3, VLife Sciences Technologies Pvt. Ltd., Pune, India

[B38] Wold S Sjöström M Eriksson L PLS-regression: a basic tool of chemometrics Chemometr Intell Lab 2001 58 2 109 130

[B39] Rucker C Rucker G Meringer M y-Randomization and its variants in QSPR/QSAR J Chem Inf Model 2007 47 6 2345 2357 17880194 10.1021/ci700157b

[B40] Schrodinger suite 2009 Schrodinger, LLC, New York, NY

[B41] Friesner RA Banks JL Murphy RB Halgren TA Klicic JJ Mainz DT Repasky MP Knoll EH Shelley M Perry JK Glide: a new approach for rapid, accurate docking and scoring. 1. Method and assessment of docking accuracy J Med Chem 2004 47 7 1739 1749 15027865 10.1021/jm0306430

[B42] Halgren TA Murphy RB Friesner RA Beard HS Frye LL Pollard WT Banks JL Glide: a new approach for rapid, accurate docking and scoring. 2. Enrichment factors in database screening J Med Chem 2004 47 7 1750 1759 15027866 10.1021/jm030644s

[B43] Sali A Blundell TL Comparative protein modelling by satisfaction of spatial restraints J Mol Biol 1993 234 3 779 815 8254673 10.1006/jmbi.1993.1626

[B44] Fiser A Do RKG Šali A Modeling of loops in protein structures Protein Sci 2000 9 9 1753 1773 11045621 10.1110/ps.9.9.1753 PMC2144714

[B45] Eswar N Webb B Marti-Renom MA Madhusudhan M Eramian D Shen My Pieper U Sali A Comparative protein structure modeling using Modeller Curr Protoc Bioinformatics 2006 5.6.1 5.6.30 10.1002/0471250953.bi0506s15 PMC4186674 18428767

[B46] Martí-Renom MA Stuart AC Fiser A Sánchez R Melo F Šali A Comparative protein structure modeling of genes and genomes Annu Rev Biophys Bio 2000 29 1 291 325 10.1146/annurev.biophys.29.1.291 10940251

[B47] Bhattacharya A Tejero R Montelione GT Evaluating protein structures determined by structural genomics consortia Proteins 2007 66 4 778 795 17186527 10.1002/prot.21165

[B48] Joosten RP Te Beek TA Krieger E Hekkelman ML Hooft RW Schneider R Sander C Vriend G A series of PDB related databases for everyday needs Nucleic Acids Res 2011 39 suppl 1 D411 D419 21071423 10.1093/nar/gkq1105 PMC3013697

[B49] Kabsch W Sander C Dictionary of protein secondary structure: pattern recognition of hydrogen-bonded and geometrical features Biopolymers 1983 22 12 2577 2637 6667333 10.1002/bip.360221211

[B50] Zimmerman DE Kulikowski CA Huang Y Feng W Tashiro M Shimotakahara S Chien C-y Powers R Montelione GT Automated analysis of protein NMR assignments using methods from artificial intelligence J Mol Biol 1997 269 4 592 610 9217263 10.1006/jmbi.1997.1052

[B51] Huang YJ Powers R Montelione GT Protein NMR recall, precision, and F-measure scores (RPF scores): structure quality assessment measures based on information retrieval statistics J Am Chem Soc 2005 127 6 1665 1674 15701001 10.1021/ja047109h

[B52] Huang YJ Tejero R Powers R Montelione GT A topology-constrained distance network algorithm for protein structure determination from NOESY data Proteins 2006 62 3 587 603 16374783 10.1002/prot.20820

[B53] Eisenberg D Lüthy R Bowie JU VERIFY3D: assessment of protein models with three-dimensional profiles Method Enzymol 1997 277 396 10.1016/s0076-6879(97)77022-8 9379925

[B54] Sippl MJ Recognition of errors in three-dimensional structures of proteins Proteins 1993 17 4 355 362 8108378 10.1002/prot.340170404

[B55] Laskowski RA MacArthur MW Moss DS Thornton JM PROCHECK: a program to check the stereochemical quality of protein structures J Appl Crystallogr 1993 26 2 283 291

[B56] Davis IW Leaver-Fay A Chen VB Block JN Kapral GJ Wang X Murray LW Arendall WB Snoeyink J Richardson JS MolProbity: all-atom contacts and structure validation for proteins and nucleic acids Nucleic Acids Res 2007 35 suppl 2 W375 W383 17452350 10.1093/nar/gkm216 PMC1933162

[B57] Sastry GM Adzhigirey M Day T Annabhimoju R Sherman W Protein and ligand preparation: parameters, protocols, and influence on virtual screening enrichments J Comput Aid Mol Des 2013 27 3 221 234 10.1007/s10822-013-9644-8 23579614

[B58] Friesner RA Murphy RB Repasky MP Frye LL Greenwood JR Halgren TA Sanschagrin PC Mainz DT Extra precision glide: docking and scoring incorporating a model of hydrophobic enclosure for protein-ligand complexes J Med Chem 2006 49 21 6177 6196 17034125 10.1021/jm051256o

[B59] Goyal M Grover S Dhanjal JK Goyal S Tyagi C Chacko S Grover A Novel natural structure corrector of ApoE4 for checking Alzheimer's disease: benefits from high throughput screening and molecular dynamics simulations Biomed Res Int 2013 2013 620793 24324968 10.1155/2013/620793 PMC3845489

[B60] Dhanjal JK Grover S Sharma S Singh A Grover A Structural insights into mode of actions of novel natural Mycobacterium protein tyrosine phosphatase B inhibitors BMC Genomics 2014 15 Suppl 1 S3 10.1186/1471-2164-15-S1-S3 PMC4046716 24564493

[B61] Dhanjal JK Goyal S Sharma S Hamid R Grover A Mechanistic insights into mode of action of potent natural antagonists of BACE-1 for checking Alzheimer's plaque pathology Biochem Biophys Res Commun 2014 443 3 1054 1059 24365147 10.1016/j.bbrc.2013.12.088

[B62] Wallace AC Laskowski RA Thornton JM LIGPLOT: a program to generate schematic diagrams of protein-ligand interactions Protein Eng 1995 8 2 127 134 7630882 10.1093/protein/8.2.127

[B63] Beckstein O Fourrier A Iorga BI Prediction of hydration free energies for the SAMPL4 diverse set of compounds using molecular dynamics simulations with the OPLS-AA force field J Comput Aid Mol Des 2014 28 3 265 276 10.1007/s10822-014-9727-1 24557853

[B64] Guo Z Mohanty U Noehre J Sawyer TK Sherman W Krilov G Probing the alpha-helical structural stability of stapled p53 peptides: molecular dynamics simulations and analysis Chem Biol Drug Des 2010 75 4 348 359 20331649 10.1111/j.1747-0285.2010.00951.x

[B65] Bowers KJ Chow E Xu H Dror RO Eastwood MP Gregersen BA Klepeis JL Kolossvary I Moraes MA Sacerdoti FD Scalable algorithms for molecular dynamics simulations on commodity clusters SC 2006 Conference, Proceedings of the ACM/IEEE: 2006 2006 IEEE 43 43

[B66] Essmann U Perera L Berkowitz ML Darden T Lee H Pedersen LG A smooth particle mesh Ewald method J Chem Phys 1995 103 19 8577 8593

[B67] Quikprop 2011 v. 3.4, Schrodinger, LLC, New York, NY

[B68] Cheng F Li W Zhou Y Shen J Wu Z Liu G Lee PW Tang Y admetSAR: a comprehensive source and free tool for assessment of chemical ADMET properties J Chem Inf Model 2012 52 11 3099 3105 23092397 10.1021/ci300367a

