# Supplementary material for: Fragment based group QSAR and molecular dynamics mechanistic studies on arylthioindole derivatives targeting the α-β interfacial site of human tubulin
Source: BMC Genomics. 2014 Dec 8;15(Suppl 9):S3. doi: 10.1186/1471-2164-15-S9-S3 (PMC4290613; doi:10.1186/1471-2164-15-S9-S3)
Supplement: Additional file 1 — Table S1 - Structures and anti-cancer activity values of arylthioindole derivatives used in this study. [file 1471-2164-15-S9-S3-S1.docx]

**Supporting information**

**Table S1:** Structures and anticancer activity values of arylthioindole derivatives used in this study.

| S.No. | Molecule | Structure | Actual activity (pIC50) | Predicted activity |
| --- | --- | --- | --- | --- |
| 1 | 16 | 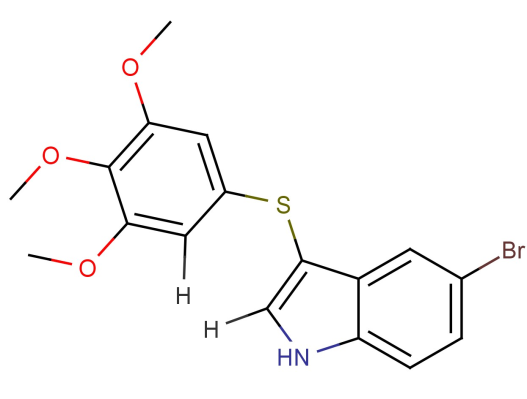 | 5.79 | 5.59086 |
| 2 | 26 | 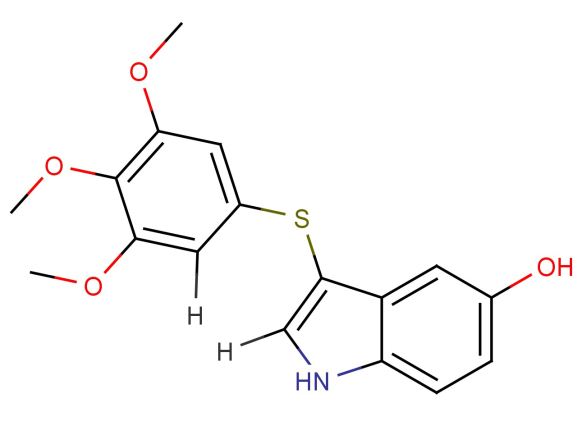 | 5.2 | 5.42972 |
| 3 | 23 | 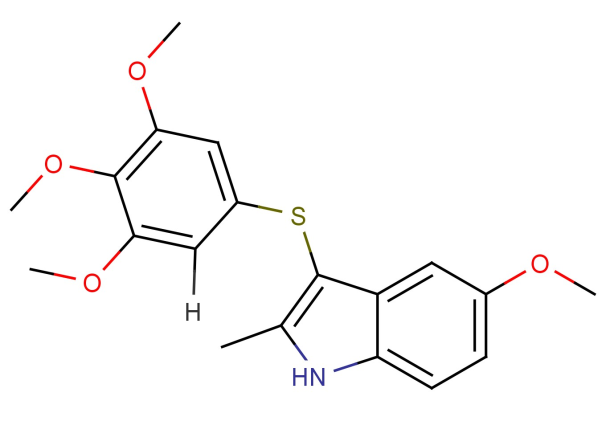 | 5.48 | 5.25563 |
| 4 | 11 | 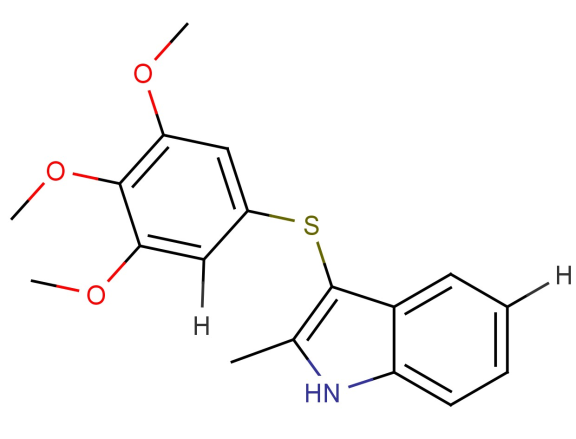 | 5.16 | 5.1787 |
| 5 | 19 | 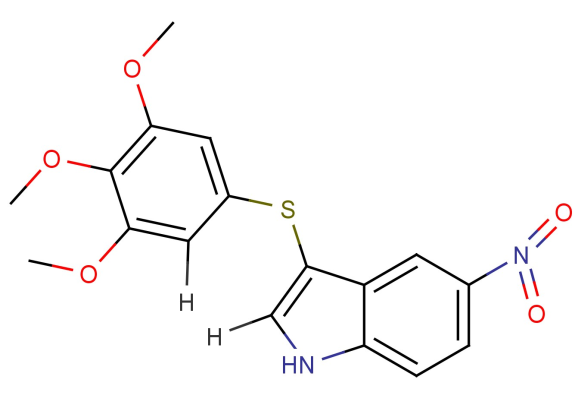 | 4.79 | 4.84748 |
| 6 | 25 | 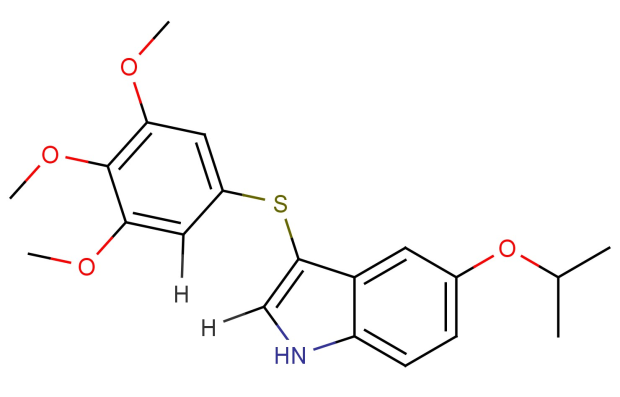 | 4.72 | 4.82379 |
| 7 | 15b | 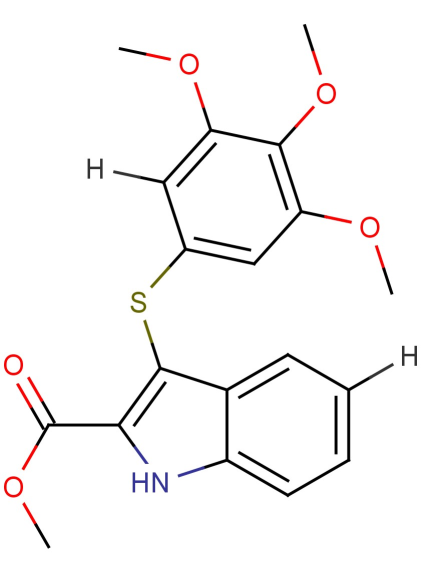 | 5.53 | 5.63467 |
| 8 | 26b | 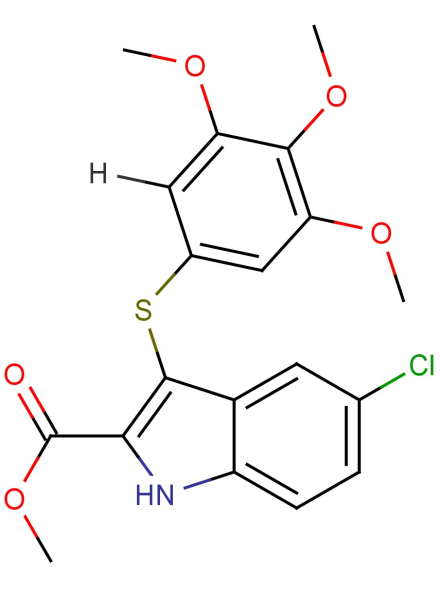 | 5.6 | 5.72292 |
| 9 | 36b | 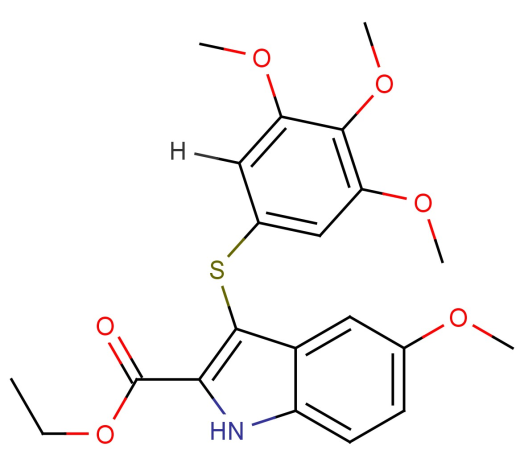 | 5.61 | 5.50157 |
| 10 | 16b | 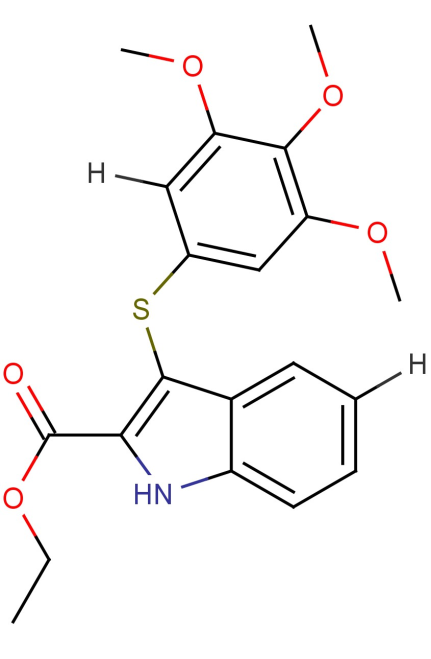 | 5.53 | 5.42464 |
| 11 | 37b | 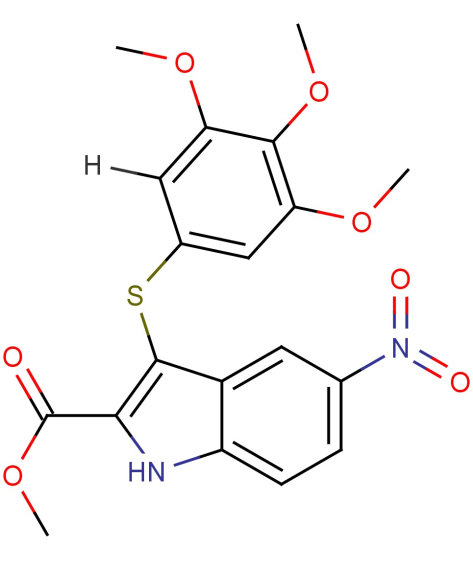 | 5.34 | 5.09342 |
| 12 | 25b | 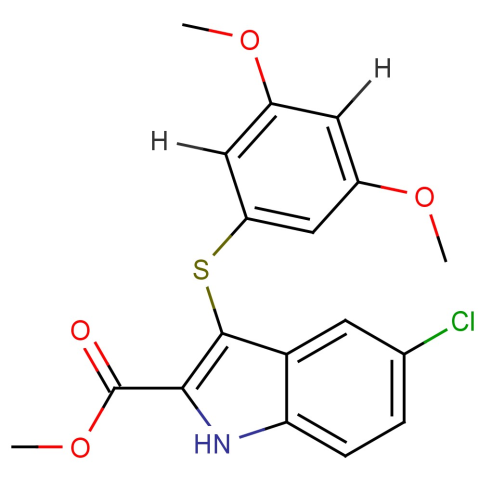 | 5.65 | 5.72292 |
| 13 | 19b | 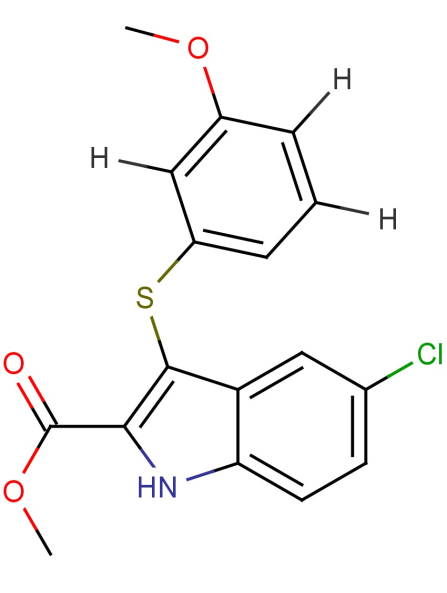 | 5.74 | 5.72292 |
| 14 | 13b | 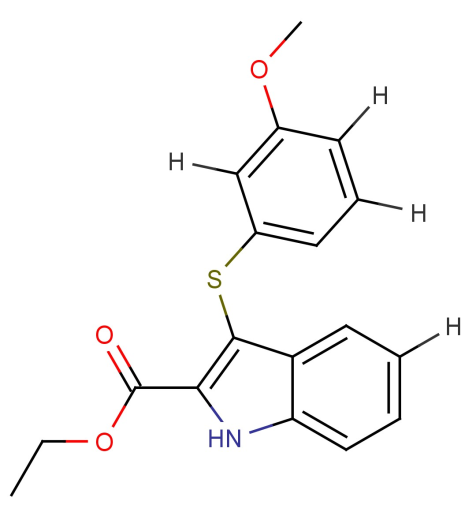 | 5.53 | 5.42464 |
| 15 | 33b | 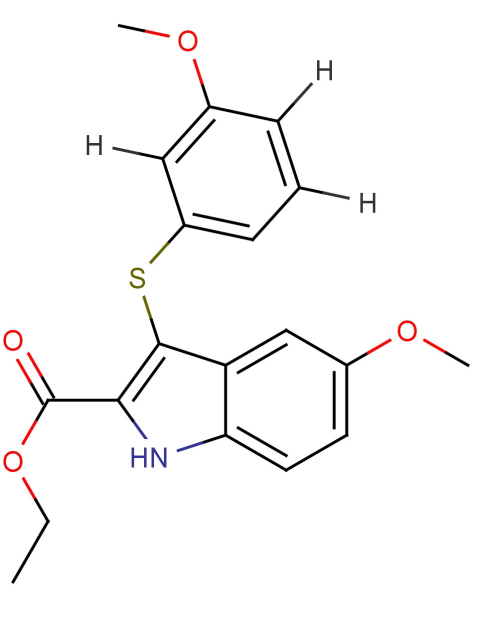 | 5.5 | 5.50157 |
| 16 | 38b | 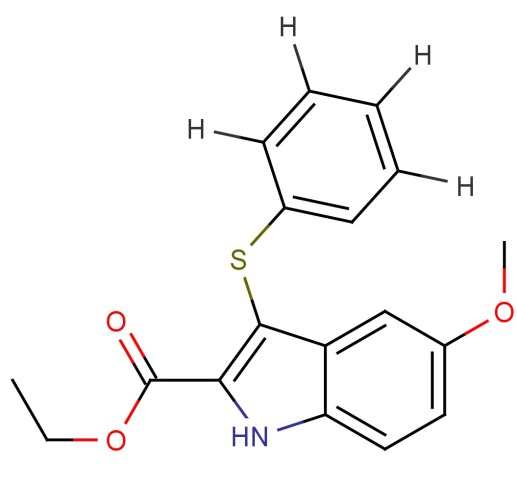 | 4.85 | 4.96478 |
| 17 | 29b | 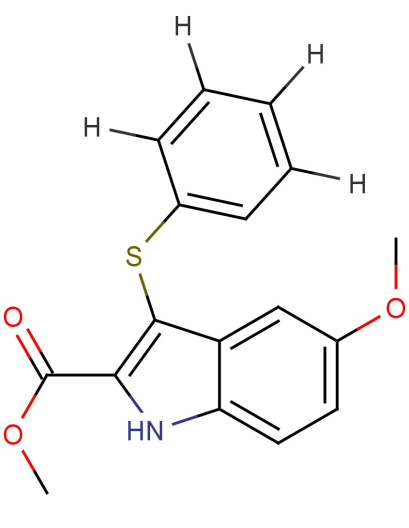 | 5.2 | 5.17481 |
| 18 | 12b | 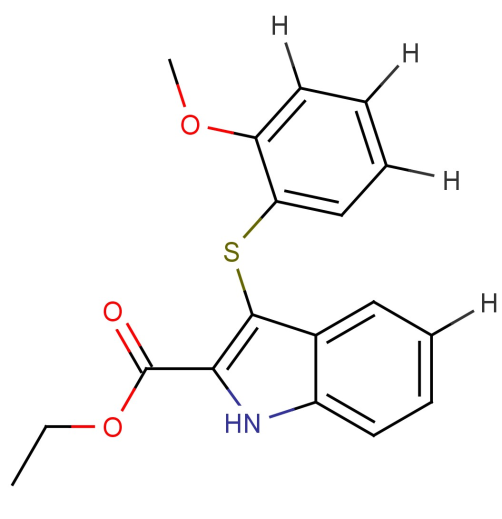 | 4.92 | 4.88785 |
| 19 | 17b | 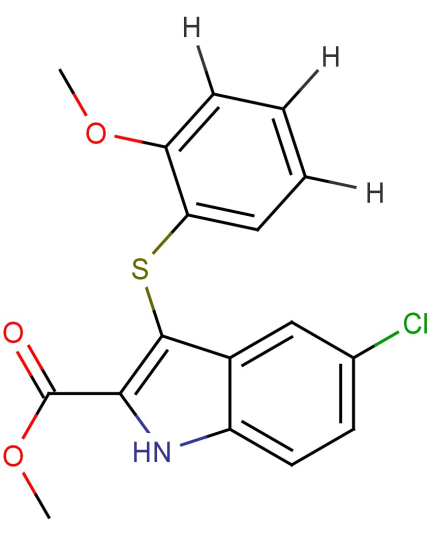 | 5.37 | 5.18613 |
| 20 | 12 | 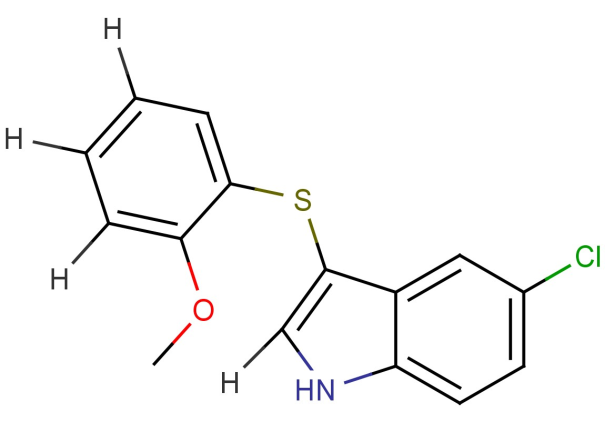 | 4.95 | 4.94019 |
| 21 | 9 | 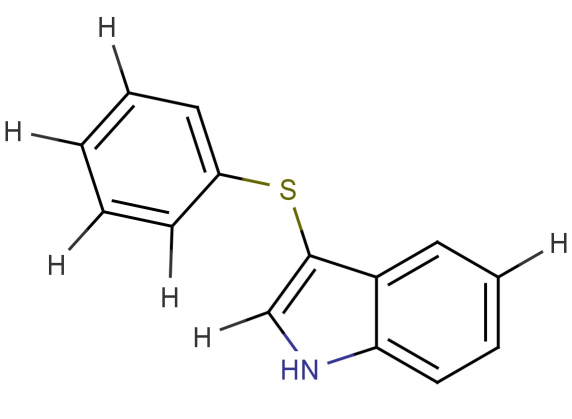 | 4.82 | 4.85194 |
| 22 | 13 | 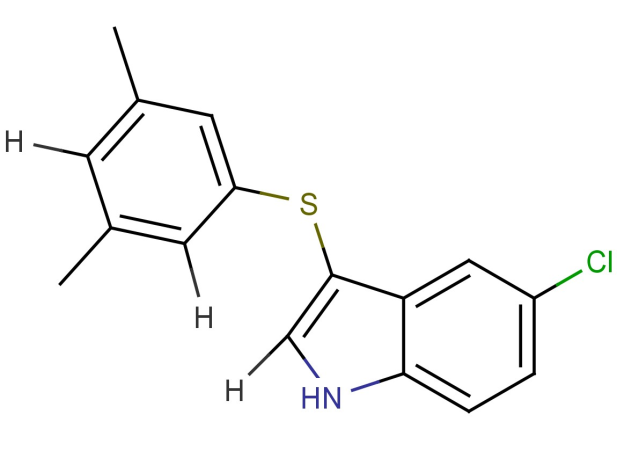 | 5.02 | 4.94019 |
| 23 | 17 | 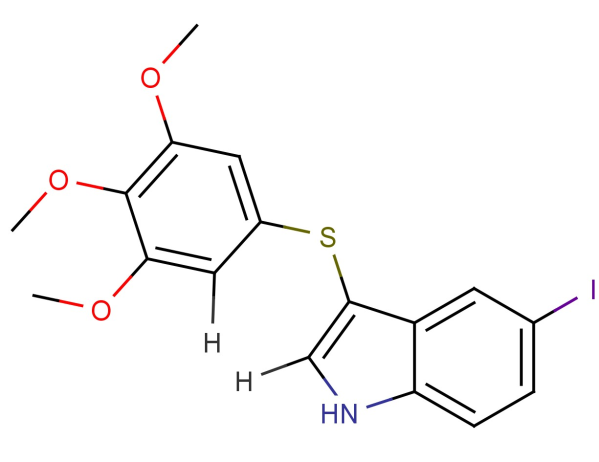 | 5.56 | 5.71129 |
| 24 | 20 | 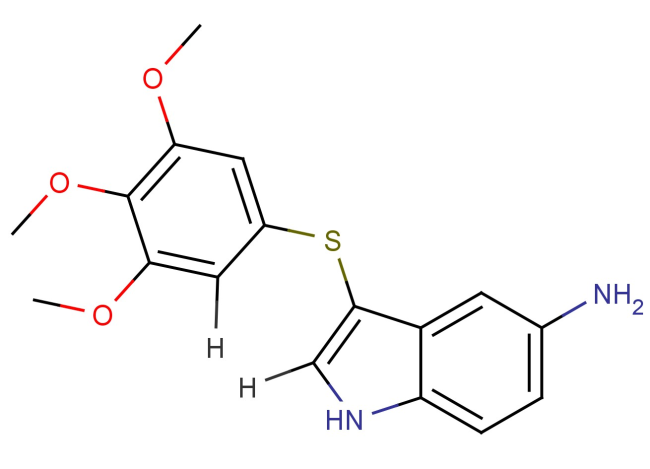 | 4.88 | 5.06205 |
| 25 | 27 | 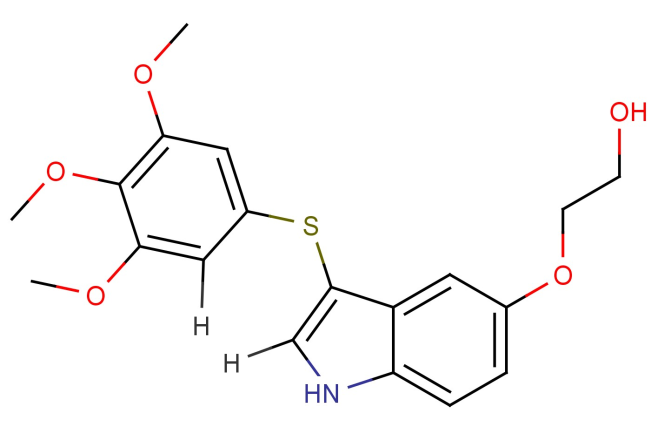 | 5.16 | 5.1305 |
| 26 | 32b | 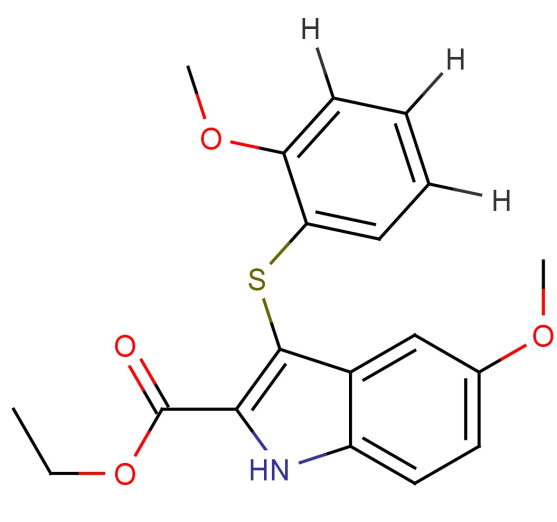 | 4.79 | 4.96478 |
| **27** | **10** | 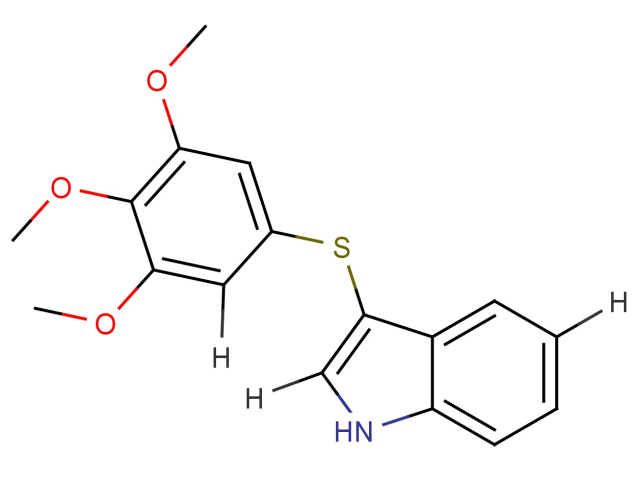 | 5.58 | 5.38873 |
| **28** | **14** | 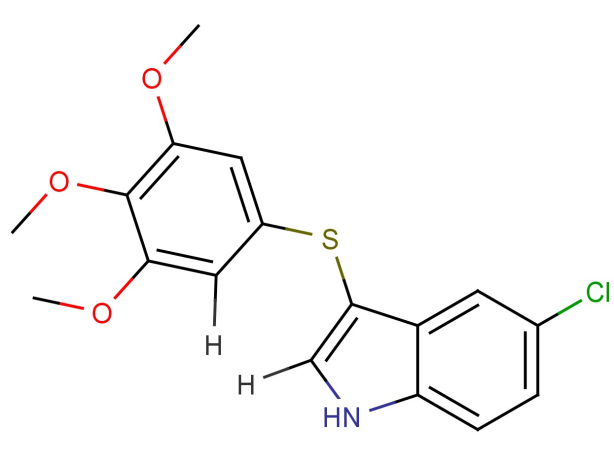 | 5.58 | 5.47698 |
| **29** | **21** | 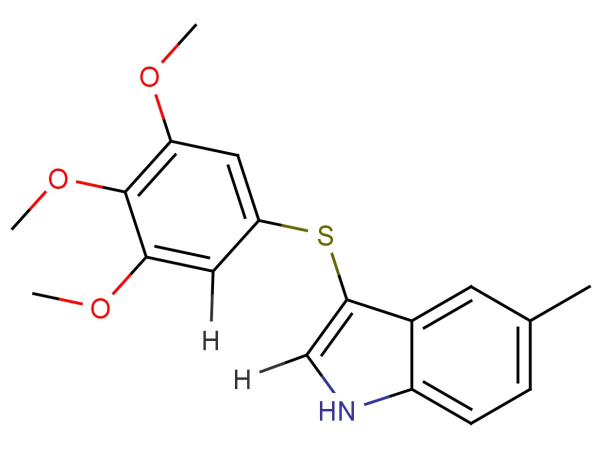 | 5.56 | 5.42467 |
| **30** | **18** | 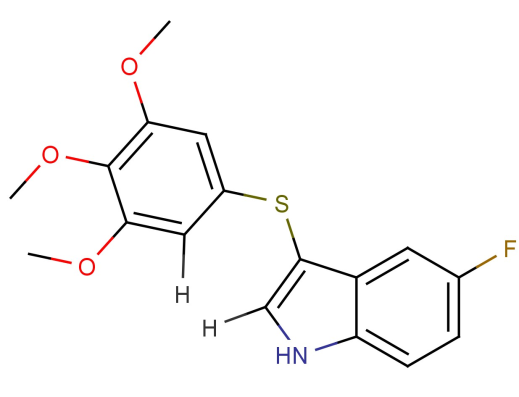 | 5.48 | 5.43482 |
| **31** | **22** | 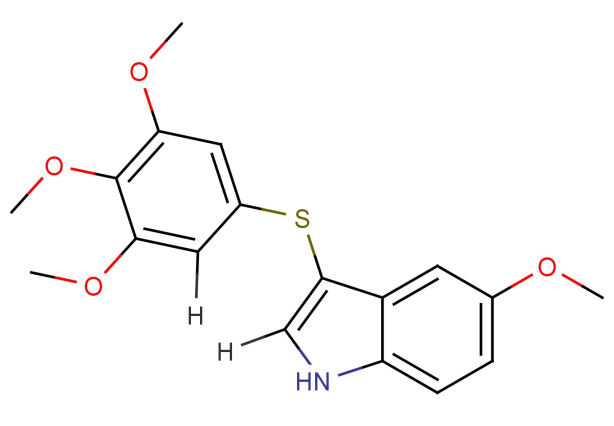 | 5.38 | 5.46566 |
| **32** | **29** | 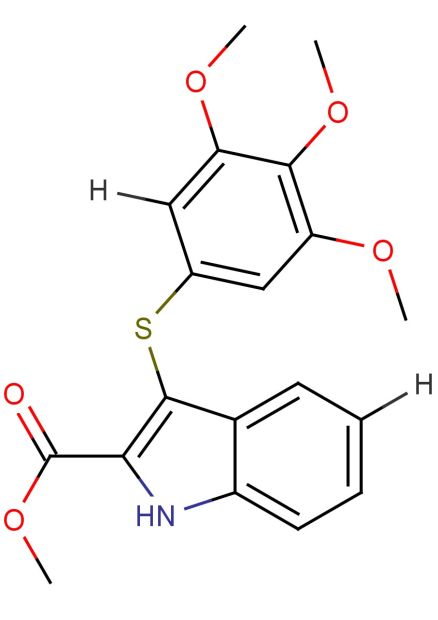 | 5.53 | 5.63467 |
| **33** | **30** | 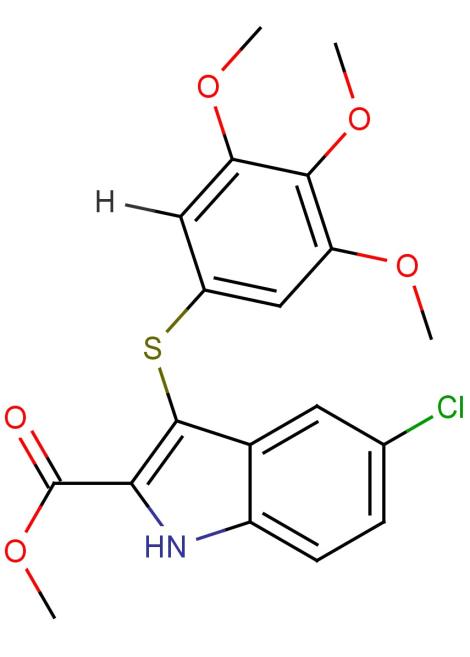 | 5.63 | 5.72292 |
| **34** | **35b** | 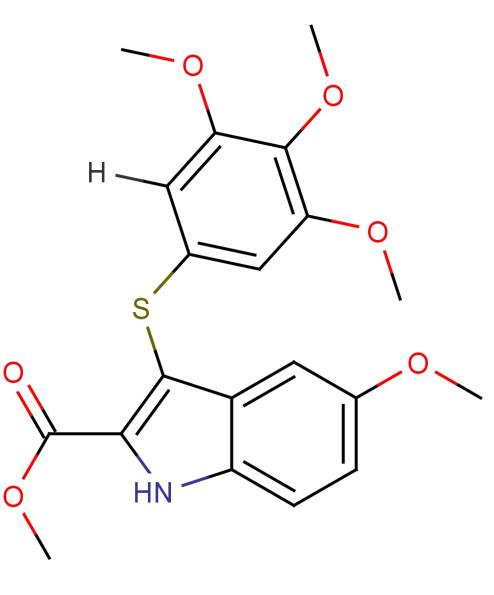 | 5.69 | 5.7116 |
| **35** | **27b** | 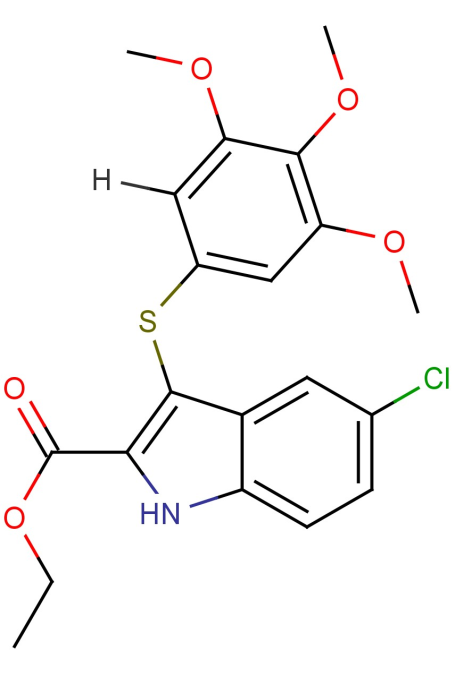 | 5.65 | 5.51289 |
| **36** | **31** | 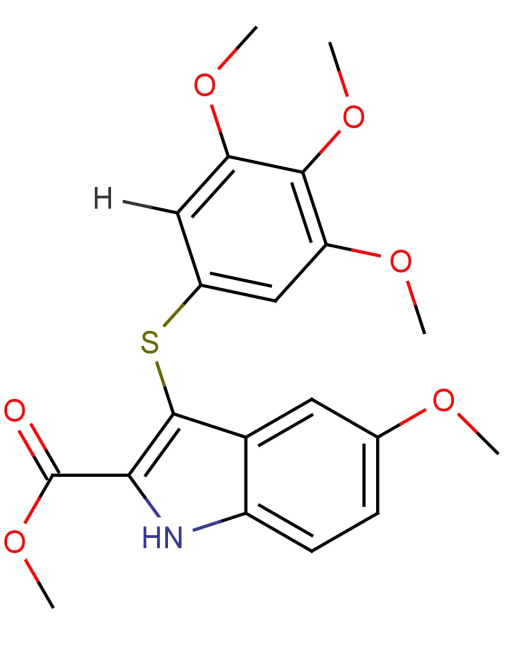 | 5.69 | 5.7116 |
