# Supplementary material for: Fragment based group QSAR and molecular dynamics mechanistic studies on arylthioindole derivatives targeting the α-β interfacial site of human tubulin
Source: BMC Genomics. 2014 Dec 8;15(Suppl 9):S3. doi: 10.1186/1471-2164-15-S9-S3 (PMC4290613; doi:10.1186/1471-2164-15-S9-S3)
Supplement: Additional file 2 — Table S2 - Calculated descriptor values and predicted activity of arylthioindole derivatives. [file 1471-2164-15-S9-S3-S2.docx]

**Additional File 2**

**Table S2.** Calculated descriptor values and predicted activity of arylthioindole derivatives.

| Arylthioindole derivatives | pIC50 | R4-H Acceptor Count | R6-chi2 | R5-slogp | R6-NitrogensCount | R6-Mol.Wt. | Predicted activity |
| --- | --- | --- | --- | --- | --- | --- | --- |
| 16 | 5.79 | 1 | 0 | 0.246 | 0 | 80.91 | 5.590 |
| 26 | 5.2 | 1 | 0 | 0.246 | 0 | 18.01 | 5.429 |
| 23 | 5.48 | 1 | 0 | 0.636 | 0 | 32.04 | 5.255 |
| 11 | 5.16 | 1 | 0 | 0.636 | 0 | 2.015 | 5.178 |
| 19 | 4.79 | 1 | 0.707 | 0.246 | 1 | 47.01 | 4.847 |
| 25 | 4.72 | 1 | 1.732 | 0.246 | 0 | 60.09 | 4.823 |
| 15b | 5.53 | 1 | 0 | -0.210 | 0 | 2.015 | 5.634 |
| 26b | 5.6 | 1 | 0 | -0.210 | 0 | 36.46 | 5.722 |
| 36b | 5.61 | 1 | 0 | 0.1793 | 0 | 32.04 | 5.501 |
| 16b | 5.53 | 1 | 0 | 0.1793 | 0 | 2.015 | 5.424 |
| 37b | 5.34 | 1 | 0.707 | -0.210 | 1 | 47.01 | 5.093 |
| 25b | 5.65 | 1 | 0 | -0.210 | 0 | 36.46 | 5.722 |
| 19b | 5.74 | 1 | 0 | -0.210 | 0 | 36.46 | 5.722 |
| 13b | 5.53 | 1 | 0 | 0.1793 | 0 | 2.015 | 5.424 |
| 33b | 5.5 | 1 | 0 | 0.1793 | 0 | 32.04 | 5.501 |
| 38b | 4.85 | 0 | 0 | 0.1793 | 0 | 32.04 | 4.964 |
| 29b | 5.2 | 0 | 0 | -0.210 | 0 | 32.04 | 5.174 |
| 12b | 4.92 | 0 | 0 | 0.246 | 0 | 2.01 | 4.887 |
| 17b | 5.37 | 0 | 0 | 0.246 | 0 | 36.46 | 5.186 |
| 12 | 4.95 | 0 | 0 | 0.246 | 0 | 36.46 | 4.940 |
| 9 | 4.82 | 0 | 0 | 0.246 | 0 | 2.015 | 4.851 |
| 13 | 5.02 | 0 | 0 | 0.246 | 0 | 36.46 | 4.940 |
| 17 | 5.56 | 1 | 0 | 0.246 | 0 | 127.9 | 5.711 |
| 20 | 4.88 | 1 | 0 | 0.246 | 1 | 17.03 | 5.062 |
| 27 | 5.16 | 1 | 1 | 0.246 | 0 | 62.06 | 5.130 |
| 32b | 4.79 | 0 | 0 | 0.1793 | 0 | 32.04 | 4.964 |
| 10 | 5.58 | 1 | 0 | 0.246 | 0 | 2.015 | 5.388 |
| 14 | 5.58 | 1 | 0 | 0.246 | 0 | 36.46 | 5.476 |
| 21 | 5.56 | 1 | 0 | 0.246 | 0 | 16.04 | 5.424 |
| 18 | 5.48 | 1 | 0 | 0.246 | 0 | 20.00 | 5.434 |
| 22 | 5.38 | 1 | 0 | 0.246 | 0 | 32.04 | 5.465 |
| 29 | 5.53 | 1 | 0 | -0.210 | 0 | 2.015 | 5.634 |
| 30 | 5.63 | 1 | 0 | -0.210 | 0 | 36.46 | 5.722 |
| 35b | 5.69 | 1 | 0 | -0.210 | 0 | 32.04 | 5.711 |
| 27b | 5.65 | 1 | 0 | 0.1793 | 0 | 36.46 | 5.512 |
| 31 | 5.69 | 1 | 0 | -0.210 | 0 | 32.04 | 5.711 |
